# Acupuncture versus tricyclic antidepressants in the prophylactic treatment of tension-type headaches: an indirect treatment comparison meta-analysis

**DOI:** 10.1186/s10194-024-01776-5

**Published:** 2024-04-29

**Authors:** Qing-Feng Tao, Yan-Bing Huang, Lu Yuan, Yun-Zhou Shi, Di Qin, Kun Ye, Wen-Yan Peng, Chao-Rong Xie, Hui Zheng

**Affiliations:** 1 The Third Hospital/Acupuncture and Tuina School, Chengdu University of Traditional Chinese Medicine, No.1166 Liutai Avenue, Wenjiang District, Chengdu, 611100 China; 2 TCM Cancer Treatment Center, Chongqing University Cancer Hospital, Chongqing, China

**Keywords:** Acupuncture, Tricyclic antidepressants, Tension-type headache, Indirect treatment comparison, Meta-analysis

## Abstract

**Background:**

Acupuncture showed better improvement than sham acupuncture in reducing attack frequency of tension-type headache (TTH), but its effectiveness relative to first-line drugs for TTH is unknown, which impedes the recommendation of acupuncture for patients who are intolerant to drugs for TTH. We aimed to estimate the relative effectiveness between acupuncture and tricyclic antidepressants (TCAs) through indirect treatment comparison (ITC) meta-analysis.

**Methods:**

We searched Ovid Medline, Embase, and Cochrane Library from database inception until April 13, 2023. Randomized controlled trials of TCAs or acupuncture in the prevention of TTH in adults were included. The primary outcome was headache frequency. The secondary outcomes were headache intensity, responder rate, and adverse event rate. Bayesian random-effect models were used to perform ITC meta-analysis, and confidence of evidence was evaluated by using the GRADE approach.

**Results:**

A total of 34 trials involving 4426 participants were included. Acupuncture had similar effect with TCAs in decreasing TTH frequency (amitriptyline: mean difference [MD] -1.29, 95% CI -5.28 to 3.02; amitriptylinoxide: MD -0.05, 95% CI -6.86 to 7.06) and reducing TTH intensity (amitriptyline: MD 2.35, 95% CI -1.20 to 5.78; clomipramine: MD 1.83, 95% CI -4.23 to 8.20). Amitriptyline had a higher rate of adverse events than acupuncture (OR 4.73, 95% CI 1.42 to 14.23).

**Conclusion:**

Acupuncture had similar effect as TCAs in reducing headache frequency of TTH, and acupuncture had a lower adverse events rate than amitriptyline, as shown by very low certainty of evidence.

**Supplementary Information:**

The online version contains supplementary material available at 10.1186/s10194-024-01776-5.

## Highlights

Acupuncture showed better improvement than sham acupuncture in reducing headache frequency of tension-type headache (TTH), but the lack of comparisons between acupuncture and first-line drugs impedes recommendation of acupuncture for TTH.

Our indirect treatment comparison meta-analysis found similar effect between acupuncture and tricyclic antidepressants (TCAs) in reducing TTH frequency with very low certainty of evidence.

Similar effect between acupuncture and TCAs were also observed in reducing headache intensity with very low certainty of evidence.

Acupuncture had a lower rate of adverse events than amitriptyline with very low certainty of evidence.

**Supplementary Information:**

The online version contains supplementary material available at 10.1186/s10194-024-01776-5.

## Background

Tension-type headache (TTH), a common neurological disease, is characterized by recurrent bilateral, tightening or pressing, and mild-to-moderate headache [1, 2]. TTH represents a crucial health issue that affects approximately 26.8% of individuals worldwide and has a female preponderance with a gender incidence ratio of 1.2:1 [3]. The condition contributes to the burden of the economy and lowers the quality of life for the people who suffer from it [3]. Acute medication and lifestyle modifications are the major methods for controlling infrequent TTH (lasting from 30 min to seven days, which occur less than once per month), however frequent episodic TTH (occur on 1–14 days per month) or chronic TTH (on 15 or more days per month) may necessitate prophylactic medications and/or behavioral therapies [1, 2].

Tricyclic antidepressants (TCAs) are recommended for patients who suffer from TTH as the primary prophylactic therapy [1, 4]. Evidence from randomized controlled trials (RCTs) and systematic reviews showed that TCAs, such as amitriptyline and clomipramine, are effective in reducing the headache frequency and intensity of TTH [5–7]. Additionally, the guideline of the European Federation of Neurological Societies suggested that due to the limitation of high side effects of pharmacological prophylactic medications, non-pharmacotherapies also deserve to be considered [4].

Acupuncture is a treatment with a long history [8]. The measure has a regulatory effect on the body by stimulating the acupoints with specific tools (such as needle) [8, 9]. Acupuncture has been applied to manage TTH, and RCTs and systematic reviews suggested acupuncture can reduce the frequency and intensity of TTH [10–13]. However, the most of previous studies of acupuncture were compared with sham acupuncture, and there is a lack of studies to compare with positive drugs in preventing TTH. The rare evidence compared with efficacious drugs might limit the use of acupuncture in the treatment of TTH, as well as hamper doctors from developing more appropriate therapeutic plans.

Indirect treatment comparison (ITC) is a method that can be used to evaluate the relative efficacy of different interventions when there is no direct comparison [14]. This approach can provide evidence of the difference in efficacy between different interventions and can help physicians to select a better therapy. In this study, we performed an ITC analysis of RCTs providing evidence for the comparison of TCAs vs. acupuncture in patients with TTH.

## Methods

We designed, performed, and reported the study according to the Preferred Reporting Items for Systematic Reviews and Meta-analyses for Network Meta-Analyses (PRISMA-NMA) guidelines [15].

### Literature search

The following databases were searched from inception to April 13, 2023: Ovid Medline, Embase, and Cochrane Library, without any restriction in the language of publication (QF-T). The search was performed using keywords and Medical Subject Heading terms associated with TTH and acupuncture or TCAs (including amitriptyline, amitriptylinoxide, clomipramine, doxepin, imipramine, amoxapine, desipramine, dibenzepine, dosulepin, lofepramine, tianeptine, trimipramine), and the search strategies were provided in eTable 1–3. We also searched clinicaltrials.gov for any potentially missing RCTs. Additionally, the reference lists of previous systematic reviews were screened for eligible studies.

### Study selection

The duplicate studies were eliminated firstly. Then, the title and abstract of the searched studies were reviewed by two independent reviewers (Q-FT and YB-H) for potential eligible research. Next, to identify the eligible RCTs further, they read the full text. The disagreements were addressed through discussion and judged by a third reviewer (HZ) finally.

### Inclusion and exclusion criteria

The RCT was considered eligible when all of the following conditions were met: (1) The study included adult patients with TTH; (2) The diagnostic standard of the study met the criteria conducted by the International Headache Society or the Ad Hoc Committee on Classification of Headache criteria of TTH; (3) The intervention of the study included acupuncture and/or TCAs; (4) The study measured and reported at least one of the following outcomes: headache frequency (the number of headache days of per month), headache intensity, responder (reduction ≥ 50% in the number of headache days of per month) rate, and adverse event rate; (6) The study was a parallel-design RCT or a crossover-design RCT with data of the first phase. The study was excluded when the research included participants with migraine unless results were presented separately for participants with TTH.

### Outcome assessments

The primary outcome was headache frequency (the changes of the number of headache days of per month). The secondary outcomes were headache intensity (the changes of the score that was measured on visual analogue scale or numeric rating scale for pain), responder rate, and adverse event rate. We evaluated the outcomes at the end of treatment.

### Data extraction

Two reviewers (YB-H and LY) independently extracted the relevant data by standardized extraction forms, involving characteristics of the eligible RCTs, details of intervention and control arm, and data of outcomes. Inconsistencies were resolved by discussion and ultimately by the decision of a third reviewer (HZ).

### Risk of bias assessment

Two reviewers (YZ-S and DQ) independently assessed the risk of bias of included RCTs by the Cochrane risk-of-bias tool (version 2) [16]. Several questions involving the following five parts were required to estimate the ROB: randomization process, deviations from intended interventions, missing outcome data, measurement of the outcome, and selection of the reported result. The risk of bias of the eligible study was rated as low, some concerns, or high risk of bias.

### The certainty of the evidence

We assessed the certainty of evidence by the Grading of Recommendations Assessment, Development, and Evaluation (GRADE) minimally contextualized framework approach that was a method designed for network meta-analysis [17]. The certainty of evidence was assessed in two levels: high (moderate to high certainty) and low (very low to low certainty) certainty. The classification of intervention was assessed into category 0, category 1, and category 2, representing among the least effective, moderately effective, and among the most effective, respectively.

### Statistical analysis

We performed the arm-based network meta-analysis (NMA) by using *multinma* package version 0.5.1 in R 4.3.1 environment and estimated the model in a Bayesian framework using *Stan* [18, 19]. We set N (0,100^2^) prior distributions for the treatment effects and study-specific intercepts and utilized half-N (5^2^) prior for the heterogeneity standard deviation of the random-effect (RE) model. The posterior total residual deviance, the number of unconstrained data points, and the deviance information criteria (DIC) of both the RE and fixed-effect (FE) models were calculated to estimate the model fit. A closer posterior total residual deviance to the number of unconstrained data points and a smaller DIC indicates a better model fit. A difference of more than 5 points suggests a significant difference [20]. We assessed the global inconsistency by drawing the dev-dev plots of the consistency model and inconsistency model, there is no evidence of inconsistency if all the points are approximately on the line of equality. Based on pooled data from included RCTs, pair-wise comparison analyses were used to assess the mean difference (MD) of continuous outcomes and the odds ratio (OR) of binary outcomes with 95% confidence intervals (CIs). For each outcome, we conducted category-level and individual-level analyses of TCAs. The heterogeneity among studies was evaluated by tau-squared, and a value of tau-squared greater than 0.36 suggested significant heterogeneity [21]. Further, the surface under the cumulative rank curve (SUCRA) value also was calculated to estimate the ranking of each intervention in the network, with a higher SUCRA value indicating a higher ranking of the intervention [22, 23].

We conducted the following sensitivity analyses for primary outcome to estimate the robustness of results: (1) excluding RCTs at high risk of bias; (2) excluding RCTs with a randomized sample size of less than 50 participants. Further, we also performed subgroup analyses in the difference of type of TTH, classification of acupuncture, and endpoint of treatment to explore the source of heterogeneity.

## Results

### Characteristics of the included RCTs

**Fig. 1:**
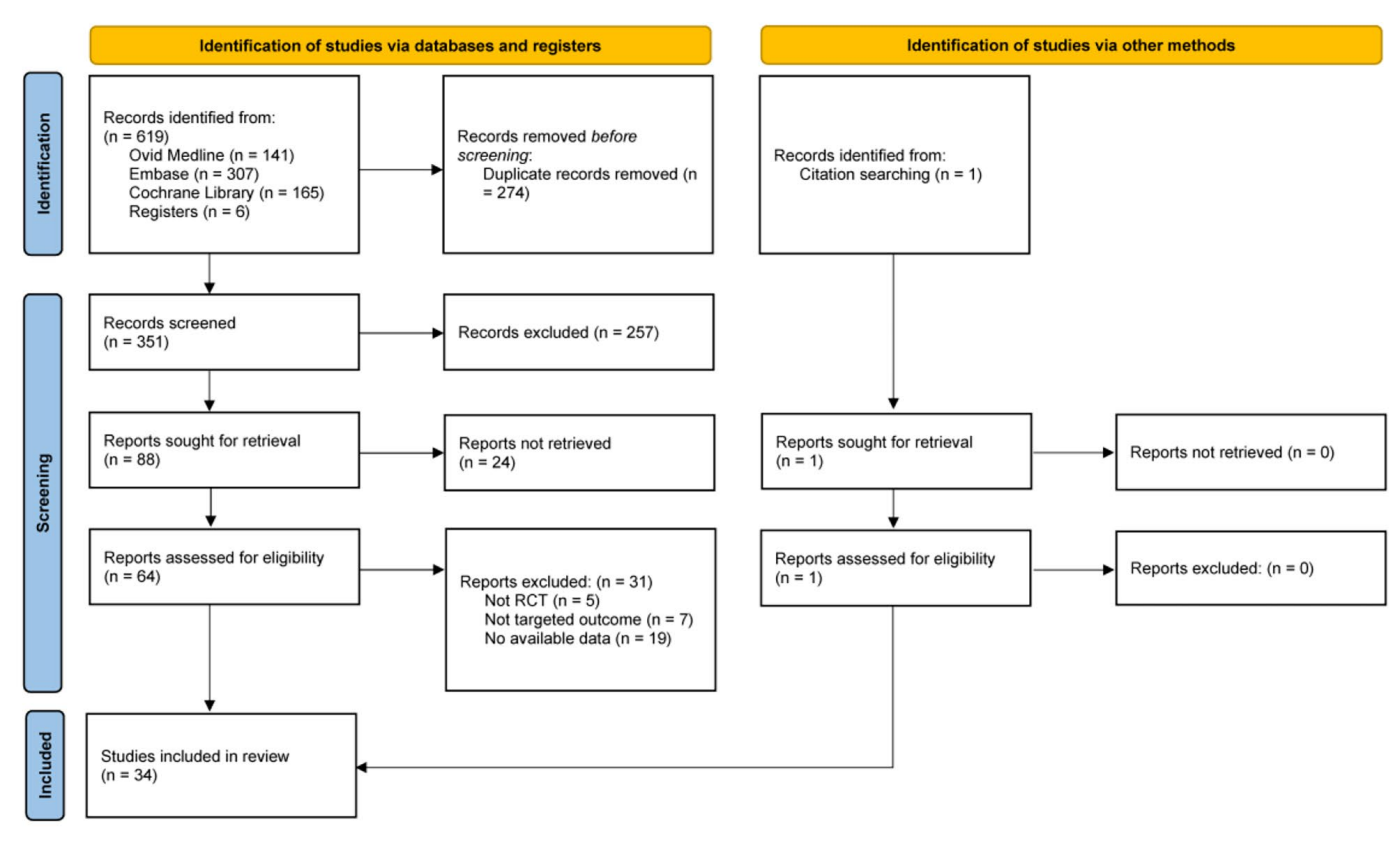
PRISMA flow diagram of literature search and study selection PRISMA, Preferred Reporting Items for Systematic Reviews and Meta-analyses

Figure 1 shows the PRISMA flowchart of literature search and study selection. Our study identified 620 articles, and after removing the duplicate articles, 351 articles were screened the titles and abstracts. Subsequently, 66 articles were reviewed in full text. Finally, 34 articles [5, 6, 10, 11, 24–53] with 4426 participants were included in our research.

Table 1 presents the characteristics of the eligible RCTs. These eligible RCTs were performed in 18 countries. The average age of included participants was 41.1 years old, and 70% of participants were female. Eighteen RCTs included acupuncture, and sixteen RCTs included TCAs, of which twelve RCTs included amitriptyline, one RCT included both amitriptyline and doxepin, one RCT included amitriptyline and amitriptylinoxide, one RCT included imipramine, and one RCT included clomipramine. The results of risk of bias assessment in eligible RCTs are presented in eFigure 1. Eight (23.53%) RCTs were evaluated as low risk of bias, eighteen (52.94%) RCTs were evaluated as some concerns, and eight (23.53%) were evaluated as high risk of bias.

**Table 1:** Characteristics of the included RCTs

Study	Country	Diagnostic criteria	Type of TTH	Interventions	Treatment duration (weeks)	Follow-up duration (weeks)	Number of patients	Female (%)	Mean ages
TCAs									
Bendtsen 1996 [42]	Denmark	IHS	Chronic	Amitriptyline 75 mg vs. placebo	8	0	40	63	40
Bettucci 2006 [47]	Italy	IHS	Chronic	Amitriptyline 20 mg vs. amitriptyline 20 mg plus tizanidine 4 mg	12	0	18	72	35.3
Boline 1995 [52]	USA	IHS	Episodic	Amitriptyline 30 mg vs. spinal manipulation	6	4	150	61	42
Boz 2003 [6]	Turkey	IHS	Chronic	Amitriptyline 25 mg vs. sertraline 50 mg	12	0	90	88	39.1
Damapong 2015 [43]	Thailand	IHS	Chronic	Amitriptyline 25 mg vs. massage	4	2	60	87	49.8
Deodato 2019 [51]	Italy	IHS	Chronic	Amitriptyline 50 mg vs. osteopathic manipulative therapy	12	0	24	60	47
Holroyd 1991 [41]	USA	IHS	Chronic	Amitriptyline 75 mg vs. cognitive-behavioral therapy	12	0	41	80	32.3
Holroyd 2001 [5]	USA	IHS	Chronic	Amitriptyline 100 mg vs. placebo	8	24	203	76	37
Indaco 1988 [45]	Italy	Ad Hoc criteria	Chronic	Amitriptyline 50 mg vs. placebo	12	0	36	52	61.2
Langemark 1990 [24]	Denmark	IHS	Chronic	Clomipramine 150 mg vs. mianserin 60 mg vs. placebo	6	0	114	NA	41
Mitsikostas 1997 [46]	Greece	IHS	Chronic	Amitriptyline 50 mg vs. buspirone 30 mg	12	0	58	62	42.5
Mousavi 2011 [48]	Iran	IHS	Chronic	Imipramine 50 mg vs. TENS	12	0	138	46	28.2
Okasha 1973 [53]	Egypt	Ad Hoc criteria	Psychogenic	Amitriptyline 30 mg vs. doxepin 30 mg vs. diazepam 6 mg vs. placebo	8	0	80	28	NA
Pfaffenrath 1994 [50]	Germany, Austria, Switzerland	IHS	Mixed	Amitriptyline 75 mg vs. placebo	12	8	197	56	38
Surbakti 2017 [49]	Indonesia	IHS	Chronic	Amitriptyline 12.5 mg vs. flunarizine 5 mg vs. flunarizine 10 mg	2	0	95	82	44.6
Vernon 2009 [44]	Canada	IHS	Chronic	Amitriptyline 25 mg plus chiropractic vs. Amitriptyline 25 mg plus sham chiropractic vs. placebo plus sham chiropractic	14	12	20	80	33.9
Acupuncture									
Chassot 2015 [36]	Brazil	IHS	Chronic	Acupuncture, twice/week vs. sham acupuncture, twice/week	5	0	34	100	40.3
Ebneshahidi 2005 [40]	Iran	IHS	Chronic	Acupuncture, 3 times/week vs. sham acupuncture, 3 times/week	4	12	50	80	35.8
Endres 2007 [29]	Germany	IHS	Mixed	Acupuncture, twice/week vs. sham acupuncture, twice/week	6	24	409	78	39.1
Gildir 2019 [26]	Turkey	IHS	Chronic	Acupuncture, 3 times/week vs. sham acupuncture, 3 times/week	2	4	160	43	36.4
Guo 2020 [32]	China	IHS	Mixed	Acupuncture, once every other day vs. eperisone hydrochloride 150 mg plus flunarizine hydrochloride	4	0	150	62	33.7
Jena 2008 [30]	Germany	IHS	Mixed	Acupuncture, 15 times/12w vs. usual care	12	12	1265	NA	NA
Karst 2001 [38]	Germany	IHS	Mixed	Acupuncture, twice/week vs. sham acupuncture, twice/week	5	20	69	55	48.1
Kawk 2007 [39]	Korea	IHS	Chronic	Acupuncture, twice/week vs. sham acupuncture, twice/week	4	12	32	NA	81
Melchart 2005 [11]	Germany	IHS	Mixed	Acupuncture, 1–2 times/week vs. sham acupuncture, 1–2 times/week vs. waiting list	8	16	270	74	42.7
Schiller 2021 [33]	Germany	IHS	Mixed	Acupuncture, twice/week vs. usual care vs. medical training therapy	6	18	72	74	38.5
Silva 2012 [28]	Brazil	IHS	Mixed	Acupuncture, 8–12 times/8week vs. usual care	8	0	43	100	27.3
Söderberg 2006 [31]	Sweden	IHS	Chronic	Acupuncture, once/week vs. physical training vs. relaxation training	10–12	24	90	81	37.4
Wang 2007 [34]	Denmark	IHS	Chronic	Acupuncture, 14 times/week vs. sham acupuncture, 14 times/week	4	6	40	50	45.3
White 1996 [25]	UK	IHS	Episodic	Acupuncture, once/week vs. sham acupuncture, once/week	6	3	10	70	57.3
White 2000 [27]	UK	IHS	Episodic	Acupuncture, once/week vs. sham acupuncture, once/week	5	12	50	76	49
Xu 2015 [35]	China	IHS	Mixed	Acupuncture, 3 times/week vs. head acupuncture, 3 times/week	4	0	60	67	NA
Xue 2004 [37]	Australia	IHS	Mixed	Acupuncture, twice/week vs. sham acupuncture, twice/week	4	0	40	65	42.1
Zheng 2022 [10]	China	IHS	Chronic	Acupuncture, 2–3 times/week vs. sham acupuncture, 2–3 times/week	8	24	218	79	43.1

RCT, randomized controlled trial; TTH, tension-type headache; TCAs, tricyclic antidepressants; IHS, International Headache Society

For all the outcomes, we compared the total posterior residual deviance with the number of unconstrained data points and DIC for all RE and FE NMA models. As the results are shown in eTable 4, the RE model had a residual deviance that was closer to the number of unconstrained data points and a lower DIC. Meanwhile, according to the dev-dev plots, all points lie roughly on the line of equality, indicating that there is no evidence for inconsistency (eFigure 2). Therefore, we choose the RE consistency models.

### Headache frequency

**Fig. 2:**
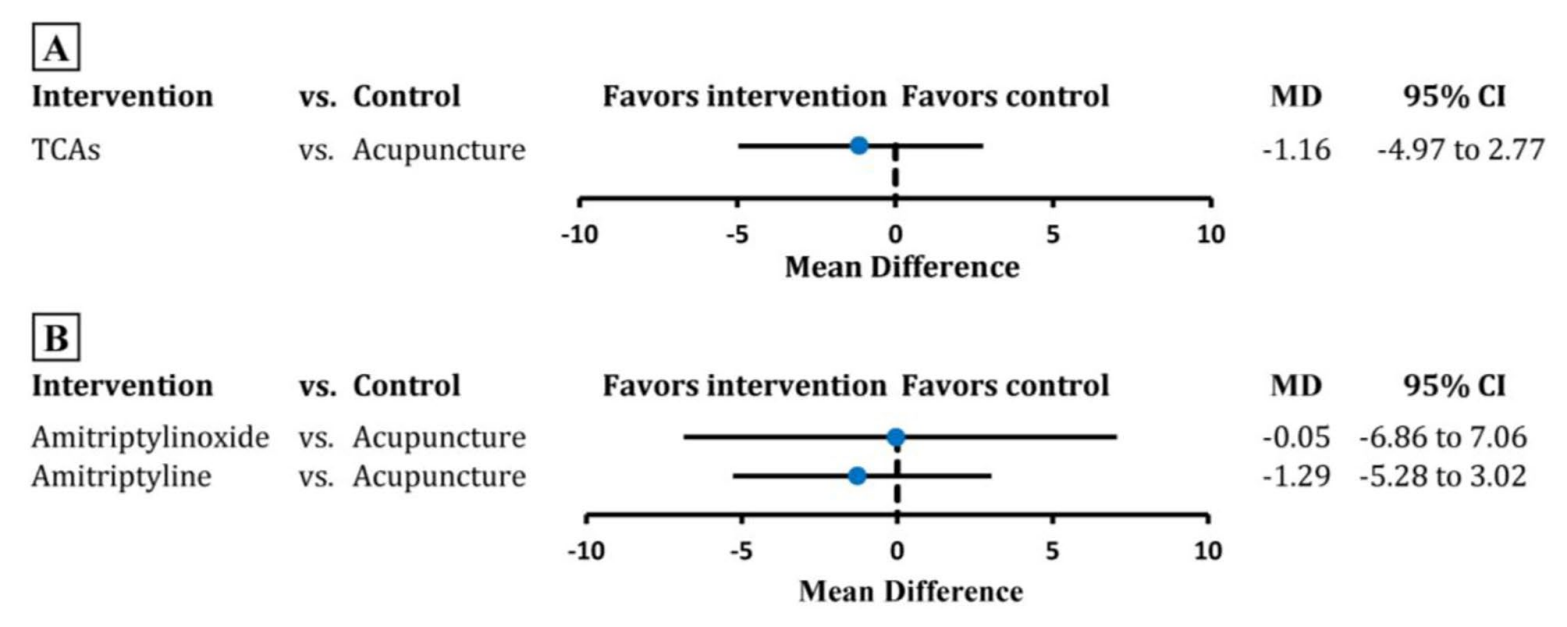
Estimate of comparison between acupuncture and TCAs of headache frequency TCAs, tricyclic antidepressants; MD, mean difference; CI, confidence interval. The black vertical line corresponds to 0。

Nineteen RCTs containing 3046 participants were pooled in the analysis on headache frequency at the end of treatment. Evidence from pair-wise comparison indicated no significant difference between TCAs and acupuncture in reducing the TTH frequency (MD -1.16, 95% CI -4.97 to 2.77; tau-squared 11.30, Fig. 2A, eFigure 3). Very low certainty of evidence showed that acupuncture was no significantly different to amitriptyline and amitriptylinoxide (amitriptyline: MD -1.29, 95% CI -5.28 to 3.02; amitriptylinoxide: MD -0.05, 95% CI -6.86 to 7.06; tau-squared 12.15; Fig. 2B, eFigure 4, Table 2, eTable 5). The SUCRA suggested amitriptyline ranked first with a value of 0.67 (eTable 6).

**Table 2:** Final classification of TCAs and acupuncture, based on NMA of intervention for headache frequency

Certainty of the evidence, and classification* of intervention	Intervention	Certainty of the evidence**
**Low certainty (low to very low certainty evidence)**		
Category 0: might be not convincingly different than acupuncture	Amitriptyline	Very low
	Amitriptylinoxide	Very low

TCAs, tricyclic antidepressants; NMA, network meta-analysis. *Categories do not inform value judgements about the importance of the effects; **Certainty of evidence for each intervention when compared with acupuncture

### Headache intensity

**Fig. 3:**
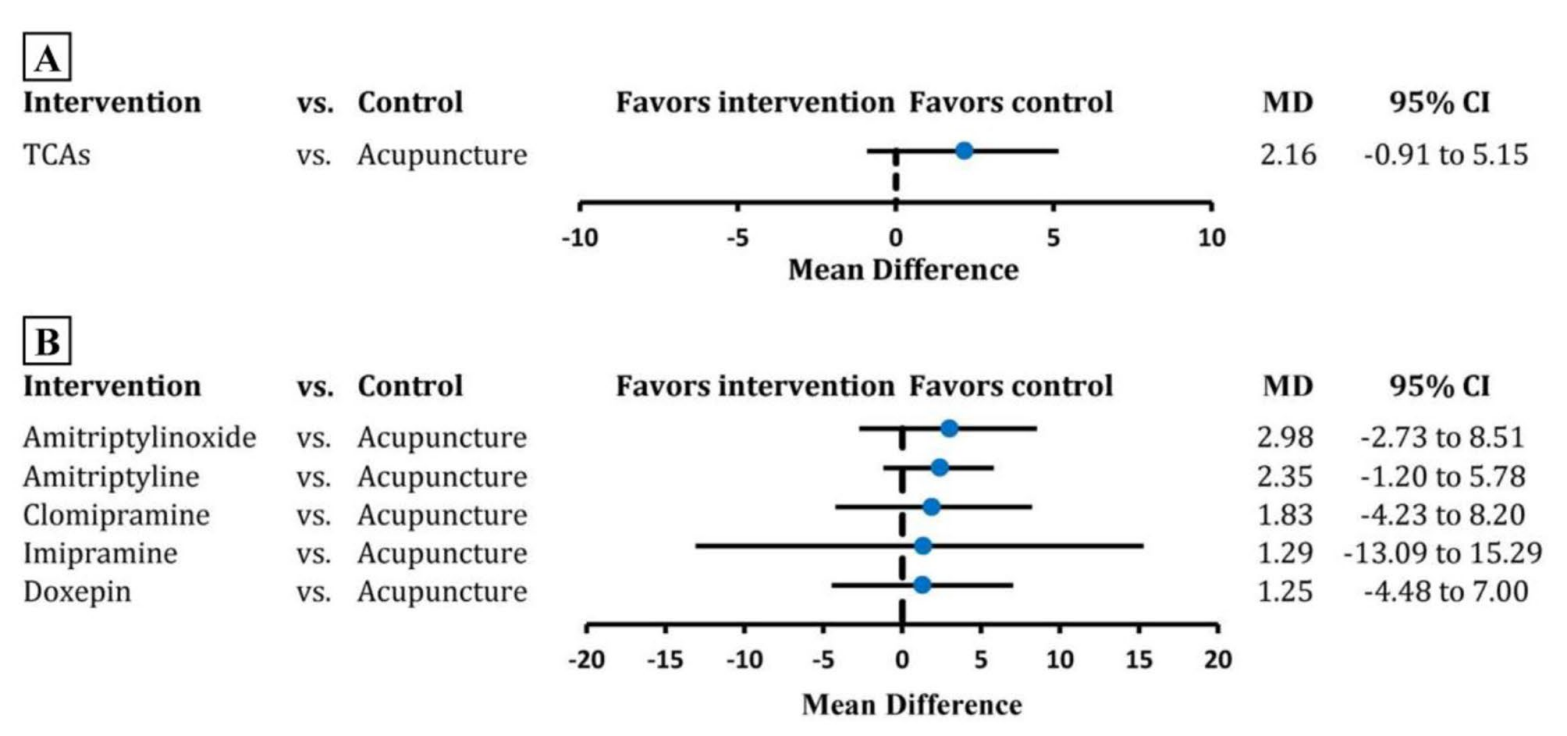
Estimate of comparison between acupuncture and TCAs of headache intensity TCAs, tricyclic antidepressants; MD, mean difference; CI, confidence interval. The black vertical line corresponds to 0

Twenty-four RCTs including 2062 individuals were entered in the analysis on headache intensity. Pair-wise comparisons revealed that acupuncture presented no significant difference with TCAs in reducing the headache intensity (MD 2.16, 95% CI -0.91 to 5.15; tau-squared 7.14; Fig. 3A, eFigure 5). At an individual-level, acupuncture presented a similar effect size with amitriptylinoxide, amitriptyline, and clomipramine with very low certainty of evidence (amitriptylinoxide: MD 2.98, 95% CI -2.73 to 8.51; amitriptyline: MD 2.35, 95% CI -1.20 to 5.78; clomipramine: MD 1.83, 95% CI -4.23 to 8.20; tau-squared 8.78; Fig. 3B, eFigure 6, eTable 5, eTable 7). The results of SUCRA showed acupuncture ranked first (SUCRA 0.77, eTable 6).

### Responder rate

Eight RCTs involving 2536 participants were included in the analysis of the responder rate. Evidence from pair-wise comparison suggested that acupuncture performs a similar effect with TCAs (OR 1.19, 95% CI 0.30 to 4.53; tau-squared 0.32; eFigure 7 A, eFigure 8). At an individual-level, there was no significant statistical difference between acupuncture and amitriptyline and amitriptylinoxide (amitriptyline, OR 0.81, 95% CI 0.16 to 4.20; amitriptylinoxide, OR 1.54, 95% CI 0.31 to 8.33; tau-squared 0.34; eFigure 7B, eFigure 9). The GRADE evidence was very low (eTable 5, eTable 8). Amitriptylinoxide ranked first with a SUCRA value of 0.89 (eTable 6).

### Adverse event rate

Nineteen RCTs containing 2052 individuals were pooled in the analysis of adverse events at the end of treatment. The main adverse events due to acupuncture were hematoma and pain; the main adverse events associated with amitriptyline were dry mouth and drowsiness. There was no significant difference between acupuncture and TCAs in adverse events rate (OR 3.37, 95% CI 0.90 to 12.99; tau-squared 0.80; eFigure 10 A, eFigure 11). At an individual-level, very low certainty of evidence suggested that amitriptyline had a significantly higher adverse event rate than acupuncture, but amitriptylinoxide was similar to acupuncture with very low GRADE evidence (amitriptyline: OR 4.73, 95% CI 1.42 to 14.23; amitriptylinoxide: OR 1.15, 95% CI 0.25 to 7.05; tau-squared 0.39; eFigure 10B, eFigure 12, eTable 5, eTable 9). The results of SUCRA suggested acupuncture ranked first (SUCRA 0.37, eTable 6).

### Sensitivity analysis

To evaluate the robustness of the results, we conducted two sensitivity analyses of the primary outcome. When RCTs at high risk of bias were excluded, we found acupuncture showed a similar effect size with TCAs in decreasing the frequency of headache (14 RCTs with 1685 participants; amitriptylinoxide: MD 0.37, 95% CI -6.67 to 7.95; amitriptyline: MD -0.80, 95% CI -6.19 to 4.69; tau-squared 14.26; eFigure 13), indicating the results were stable. After excluding RCTs with a randomized sample size of less than 50 participants, acupuncture also presented a similar effect with TCAs (13 RCTs with 2886 participants; amitriptylinoxide: MD -1.05, 95% CI -6.26 to 3.86; amitriptyline: MD -2.18, 95% CI -5.63 to 1.26; tau-squared 5.56; eFigure 14), suggesting the results were stable.

### Subgroup analysis

We performed subgroup analyses of the primary outcome in the subtype of TTH, classification of acupuncture, and different endpoints of treatment to explore the source of heterogeneity. We found these elements did not significantly influence the heterogeneity (eFigure 15–17). Meanwhile, we also found no significant difference between acupuncture and TCAs in reducing the frequency of chronic TTH (14 RCTs with 1130 participants, amitriptylinoxide: MD -0.88, 95% CI -6.94 to 5.24; amitriptyline: MD -2.18, 95% CI -6.47 to 2.11; tau-squared 7.78; eFigure 15). In addition, there was no statistical difference of the effectiveness between acupuncture and TCAs in managing TTH when subgroup analyses were performed with acupuncture classification (eFigure 16) and endpoints of treatment (eFigure 17).

## Discussion

### Main finding

In our ITC meta-analyses, we found very low evidence demonstrating similar effectiveness of acupuncture and TCAs in preventing TTH attacks. There was no significant statistical difference between acupuncture and TCAs in reducing frequency and intensity, and in responder rate. We found amitriptyline had a higher rate of adverse events than acupuncture with an OR of 4.73 (95% CI 1.42 to 14.23).

Our results showed no significant variation between acupuncture and TCAs, which may be attributed to the fact that both interventions are effective in preventing TTH. TCAs are the first-line drugs that have been recommended for preventing TTH [4, 54]. Amitriptyline was the first-choice drug for preventing TTH and has been assessed as evidence of level A [4, 55]. Acupuncture was also effective in managing TTH [10]. As the results of previous systematic review and meta-analysis [12, 56], both TCAs (SMD 1.29) and acupuncture (SMD − 1.49) had a high effect size in reducing the number of headache days per month compared to placebo or sham acupuncture [57]. Moreover, TCAs (OR 1.41) and acupuncture (OR 1.29) presented a similar high effect size in decreasing at least 50% of headache frequency per mouth [13, 56]. Therefore, acupuncture was presented as effective as TCAs with no statistically significant difference.

Evidence from studies of TCAs and acupuncture also demonstrated that they could exert analgesic effects through various mechanisms, respectively. Amitriptyline may exert analgesic effects by inhibiting norepinephrine reuptake, antagonizing n-methyl-d-aspartate receptors, blocking muscarinic receptors and ion channels, and modulating noradrenergic and serotonergic downstream pain inhibitory systems [58, 59]. Additionally, amitriptyline can prevent TTH attacks by relieving the central sensitization [60]. The mechanisms of acupuncture analgesia involve signal molecules such as adenosine [61], γ-aminobutyric acid [62], serotonin [63], opioid peptide [64], and endocannabinoid [65]. Furthermore, recent studies have shown that acupuncture can also exert analgesic effects by recruiting β-END-containing ICAM-1 + /CD11b + immune cells [66].

Furthermore, we found amitriptyline presented a higher adverse event rate than acupuncture (OR 4.73), and the value of SUCRA also suggested amitriptyline had the lowest probability of being safe (SUCRA 0.08). Previous studies demonstrated that acupuncture is a safe therapy with few adverse events [13]. Adverse events associated with acupuncture were pain and hematoma near the needle, these symptoms were usually transient and mild. In contrast, compared with placebo, amitriptyline was more likely to contribute to dry mouth and drowsiness [56]. Acupuncture seems to be safer than amitriptyline.

### Implication for practice and research

Based on our ITC analyses, acupuncture was as effective as first-line positive drugs in decreasing TTH frequency and intensity, and had lower adverse events rate than amitriptyline. Therefore, acupuncture can be an alternative therapy for managing TTH, especially for patients who are reluctant to take medications and who have severe adverse reactions to them. Meanwhile, it is necessary to conduct head-to-head trials between acupuncture and positive drugs. Our study was an indirect comparison analysis, and we found no head-to-head study had compared the effect of acupuncture and positive drugs. Endres and colleagues attempted to design an RCT in which a proportion of participants were randomly assigned to take amitriptyline [29]. However, due to participants unwilling to receive amitriptyline, the arm was dropped. Hence head-to-head studies still should be conducted for more evidence of the effectiveness of acupuncture.

### Limitations of the study

Our study also had some limitations that should be noted when interpreting the findings. First, we only searched three specific databases and clinicaltrials.gov, and we excluded 24 studies due to the unavailability of full-text copies. Therefore, there may be relevant literature that was not included. However, we tried to avoid having eligible articles overlooked by scanning previous studies. Second, we evaluated the relative effectiveness between acupuncture and TCAs by ITC meta-analyses, and head-to-head trials are still needed to obtain direct evidence. Third, eight of thirteen-four included RCTs were assessed at high risk of bias, which might affect the quality and stability of our results. Therefore, we excluded the high risk of bias RCTs to verify the robustness of our results. Fourth, the studies we included were highly heterogeneous, and we attempted to explore the sources of heterogeneity through subgroup analyses of the TTH subtype, acupuncture classification, and endpoint of treatment. However, we found that these factors did not significantly contribute to the heterogeneity.

## Conclusions

Our indirect comparison meta-analysis suggested very low evidence that acupuncture was as effective as TCAs in reducing the frequency and intensity of TTH, and acupuncture had a lower rate of adverse events than amitriptyline. Acupuncture can be an alternative option for TTH, and head-to-head studies are warranted for more direct evidence in the future.

### Electronic supplementary material

Below is the link to the electronic supplementary material.

Supplementary Material 1

Supplementary Material 2

## Data Availability

The data that supports our study are shown in the article and supplementary material, further inquiries can be directed to the corresponding author.

## Abbreviations

CI: confidence interval
DIC: deviance information criteria
FE: fixed-effect
GRADE: Grading of Recommendations Assessment, Development, and Evaluation
ITC: Indirect treatment comparison
MD: mean difference
NMA: network meta-analysis
OR: odds ratio
PRISMA-NMA: Preferred Reporting Items for Systematic Reviews and Meta-analyses for Network Meta-Analyses
RCT: randomized controlled trial
RE: random-effect
TCA: Tricyclic antidepressant
TTH: Tension-type headache
