# Supplementary material for: Acupuncture versus tricyclic antidepressants in the prophylactic treatment of tension-type headaches: an indirect treatment comparison meta-analysis
Source: J Headache Pain. 2024 Apr 29;25(1):67. doi: 10.1186/s10194-024-01776-5 (PMC11057108; doi:10.1186/s10194-024-01776-5)
Supplement: Supplementary file 2 — Supplementary Material 2 [file 10194_2024_1776_MOESM2_ESM.docx]

**Supplementary materials files**

eTable 1. Search strategy in Ovid Medline 4

eTable 2. Search strategy in Embase 6

eTable 3. Search strategy in Cochrane Library 8

eTable 4. Summary of fixed-effects and random-effects model fit statistics from network meta-analysis 10

eTable5. Certainty of evidence assessment of outcomes 12

eTable 6. Value of SUCRA for each treatment on outcomes 15

eTable 7. Final classification of TCAs and acupuncture, based on NMA of intervention for headache intensity 16

eTable 8. Final classification of TCAs and acupuncture, based on NMA of intervention for responder rate 17

eTable 9. Final classification of TCAs and acupuncture, based on NMA of intervention for AE rate 18

eFigure 1. Risk of bias of eligible RCTs 19

eFigure 2. The “dev-dev” plots of random-effects consistency and inconsistency models for NMA by outcomes 20

eFigure 3. Network diagram of comparison of headache frequency at a category-level 21

eFigure 4. Network diagram of comparison of headache frequency at an individual-level 22

eFigure 5. Network diagram of comparison of headache intensity at a category-level 23

eFigure 6. Network diagram of comparison of headache intensity at an individual-level 24

eFigure 8. Network diagram of comparison of responder rate at a category-level 26

eFigure 9. Network diagram of comparison of responder rate at an individual-level 27

eFigure 10. Estimate of comparison between acupuncture and TCAs of adverse event rate 28

eFigure 11. Network diagram of comparison of adverse event rate at a category-level 29

eFigure 12. Network diagram of comparison of adverse event rate at an individual-level 30

eFigure 13. Sensitivity analysis of headache frequency after excluding RCTs at high risk of bias 31

eFigure 14. Sensitivity analysis of headache frequency after excluding RCTs less than 50 participants 32

eFigure 15. Subgroup analysis of headache frequency on chronic tension-type headache 33

eFigure 16. Subgroup analysis of headache frequency on different classification of acupuncture 34

eFigure 17. Subgroup analysis of headache frequency on different endpoint of treatment 35

**eTable 1.** Search strategy in Ovid Medline

| **ID** | **Search strategy** |
| --- | --- |
| 1 | randomized controlled trial.pt. |
| 2 | controlled clinical trial.pt. |
| 3 | randomized.ab,ti. |
| 4 | randomised.ab,ti. |
| 5 | placebo.ab,ti. |
| 6 | randomly.ab,ti. |
| 7 | trial.ab,ti. |
| 8 | groups.ab,ti. |
| 9 | 1 or 2 or 3 or 4 or 5 or 6 or 7 or 8 |
| 10 | limit 9 to humans |
| 11 | exp tension-type headache/ |
| 12 | tension-type headache.ab,ti. |
| 13 | 11 or 12 |
| 14 | exp acupuncture therapy/ |
| 15 | (acupunct$ or electroacupunct$ or electro-acupunct$).ab,ti. |
| 16 | 14 or 15 |
| 17 | (antidepressant* or antidepressive*).ab,ti |
| 18 | (amitriptylin* OR amoxapin* OR chlorimipramin* OR clomipramin* OR desipramin* OR dibenzepin* OR dosulepin* OR dothiepin* OR doxepin* OR imipramin* OR lofepramin* OR nortriptylin* OR tianeptin* OR trimipramin*).ab,ti. |
| 19 | 17 or 18 |
| 20 | 10 and 13 and 16 |
| 21 | 10 and 13 and 19 |
| 22 | 20 or 21 |

**eTable 2.** Search strategy in Embase

| **ID** | **Search strategy** |
| --- | --- |
| 1 | 'randomized controlled trial'/exp |
| 2 | 'controlled clinical trial'/exp |
| 3 | randomized:ab,ti |
| 4 | randomised:ab,ti |
| 5 | placebo:ab,ti |
| 6 | randomly:ab,ti |
| 7 | trial:ab,ti |
| 8 | groups:ab,ti |
| 9 | #1 OR #2 OR #3 OR #4 OR #5 OR #6 OR #7 OR #8 |
| 10 | (#1 OR #2 OR #3 OR #4 OR #5 OR #6 OR #7 OR #8) AND [humans]/lim |
| 11 | 'tension-type headache'/exp |
| 12 | 'tension-type headache':ab,ti |
| 13 | #11 OR #12 |
| 14 | 'acupuncture therapy'/exp |
| 15 | acupuncture:ab,ti OR electroacupuncture:ab,ti OR 'electro acupuncture':ab,ti |
| 16 | #14 OR #15 |
| 17 | antidepressant*:ab,ti OR antidepressive*:ab,ti |
| 18 | amitriptylin*:ab,ti OR amoxapin*:ab,ti OR chlorimipramin*:ab,ti OR clomipramin*:ab,ti OR desipramin*:ab,ti OR dibenzepin*:ab,ti OR dosulepin*:ab,ti OR dothiepin*:ab,ti OR doxepin*:ab,ti OR imipramin*:ab,ti OR lofepramin*:ab,ti OR nortriptylin*:ab,ti OR tianeptin* :ab,ti OR trimipramin*:ab,ti |
| 19 | #17 OR #18 |
| 20 | #10 AND #13 AND #16 |
| 21 | #10 AND #13 AND #19 |
| 22 | #20 OR #21 |

**eTable 3.** Search strategy in Cochrane Library

| **ID** | **Search strategy** |
| --- | --- |
| 1 | (randomized controlled trial):pt |
| 2 | (controlled clinical trial):pt |
| 3 | (randomized):ti,ab,kw |
| 4 | (randomised):ti,ab,kw |
| 5 | (placebo):ti,ab,kw |
| 6 | (randomly):ti,ab,kw |
| 7 | (trial):ti,ab,kw |
| 8 | (groups):ti,ab,kw |
| 9 | #1 OR #2 OR #3 OR #4 OR #5 OR #6 OR #7 OR #8 in Trials |
| 10 | MeSH descriptor: [Tension-Type Headache] explode all trees |
| 11 | (tension-type headache):ti,ab,kw |
| 12 | #11 OR #12 |
| 13 | MeSH descriptor: [Acupuncture Therapy] explode all trees |
| 14 | (acupunct$ OR electroacupunct$ OR electro‐acupunct$):ti,ab,kw |
| 15 | #13 OR #14 |
| 16 | (antidepressant* OR antidepressive*):ti,ab,kw |
| 17 | (amitriptylin* OR amoxapin* OR chlorimipramin* OR clomipramin* OR desipramin* OR dibenzepin* OR dosulepin* OR dothiepin* OR doxepin* OR imipramin* OR lofepramin* OR nortriptylin* OR tianeptin* OR trimipramin*):ti,ab,kw |
| 18 | #16 OR #17 |
| 19 | #9 AND #12 AND #15 |
| 20 | #9 AND #12 AND #18 |
| 21 | #19 OR #20 |

**eTable 4.** Summary of fixed-effects and random-effects model fit statistics from network meta-analysis

| **Model** | **Number of data points** | **Posterior total residual deviance** | **DIC** |
| --- | --- | --- | --- |
| **Headache frequency** | | | |
| **FE consistency** | 43 | 472 | 503.8 |
| **FE inconsistency** | 43 | 471.7 | 503.4 |
| **RE consistency** | 43 | 42.9 | 84.4 |
| **Headache intensity** | | | |
| **FE consistency** | 57 | 600.5 | 646.1 |
| **FE inconsistency** | 57 | 600.4 | 646 |
| **RE consistency** | 57 | 57.2 | 113.4 |
| **Responder rate** | | | |
| **FE consistency** | 18 | 19.9 | 34.1 |
| **FE inconsistency** | 18 | 19.8 | 33.9 |
| **RE consistency** | 18 | 18.2 | 34.8 |
| **Adverse event rate** | | | |
| **FE consistency** | 41 | 43.9 | 74.6 |
| **FE inconsistency** | 41 | 43.5 | 74 |
| **RE consistency** | 41 | 39.8 | 73.9 |

**Footnote:** DIC: deviance information criteria; FE: fixed-effects; RE: random-effects.

**eTable5. Certainty of evidence assessment of outcomes**

| **Outcome** | **N of studies** | **Study design** | **Risk of bias** | **Inconsistency** | **Indirectness** | **Imprecision** | **Publication bias** | **Certainty Evidence** | **Importance of outcome** |
| --- | --- | --- | --- | --- | --- | --- | --- | --- | --- |
| **Acupuncture vs. amitriptyline** | | | | | | | | | |
| Headache frequency | 19 | RCT | Very serious ^a^ | Serious ^b^ | Serious ^c^ | No serious | No serious | ÅOOO VERY LOW | CRITICAL |
| Headache intensity | 22 | RCT | Very serious ^a^ | Serious ^b^ | Serious ^c^ | No serious | No serious | ÅOOO VERY LOW | IMPORTANT |
| Responder rate | 8 | RCT | Very serious ^a^ | No serious | Serious ^c^ | No serious | No serious | ÅOOO VERY LOW | IMPORTANT |
| Adverse event rate | 19 | RCT | Very serious ^a^ | Serious ^b^ | Serious ^c^ | No serious | No serious | ÅOOO VERY LOW | IMPORTANT |
| **Acupuncture vs. amitriptylinoxide** | | | | | | | | | |
| Headache frequency | 11 | RCT | Very serious ^a^ | Serious ^b^ | Serious ^c^ | No serious | No serious | ÅOOO VERY LOW | CRITICAL |
| Headache intensity | 12 | RCT | Very serious ^a^ | Serious ^b^ | Serious ^c^ | No serious | No serious | ÅOOO VERY LOW | IMPORTANT |
| Responder rate | 7 | RCT | Very serious ^a^ | No serious | Serious ^c^ | No serious | No serious | ÅOOO VERY LOW | IMPORTANT |
| Adverse event rate | 12 | RCT | Very serious ^a^ | Serious ^b^ | Serious ^c^ | No serious | No serious | ÅOOO VERY LOW | IMPORTANT |
| **Acupuncture vs.** **clomipramine** | | | | | | | | | |
| Headache intensity | 12 | RCT | Very serious ^a^ | Serious ^b^ | Serious ^c^ | No serious | No serious | ÅOOO VERY LOW | IMPORTANT |
| **Acupuncture vs. doxepin** | | | | | | | | | |
| Headache intensity | 12 | RCT | Very serious ^a^ | Serious ^b^ | Serious ^c^ | No serious | No serious | ÅOOO VERY LOW | IMPORTANT |
| **Acupuncture vs. imipramine** | | | | | | | | | |
| Headache intensity | 12 | RCT | Very serious ^a^ | Serious ^b^ | Serious ^c^ | No serious | No serious | ÅOOO VERY LOW | IMPORTANT |

Footnote: RCT, randomized controlled trial.

^a^ We downgraded by two level because of there were some RCTs were assessed at high risk of bias.

^b^ We downgraded by one level because of there were high heterogeneity between studies (tau-squared > 0.36).

^c^ We downgraded by one level because of there was Indirect comparison.

**eTable 6.** **Value of SUCRA for each treatment on outcomes**

| **Treatment** | **SUCRA** | | | |
| --- | --- | --- | --- | --- |
|  | **Headache frequency** | **Headache intensity** | **Responder rate** | **Adverse event rate** |
| Acupuncture | 0.54 | 0.77 | 0.75 | 0.37 |
| Amitriptyline | 0.67 | 0.47 | 0.62 | 0.08 |
| Amitriptylinoxide | 0.53 | 0.41 | 0.89 | 0.36 |
| Clomipramine | NA | 0.55 | NA | NA |
| Doxepin | NA | 0.60 | NA | NA |
| Imipramine | NA | 0.58 | NA | NA |

**Footnote:** SUCRA: surface under the cumulative rank curve; NA: not applicable. Values nearest 1 indicate preferred treatment.

**eTable 7.** Final classification of TCAs and acupuncture, based on NMA of intervention for headache intensity

| **Certainty of the evidence, and classification* of intervention** | **Intervention** | **Certainty of the evidence**** |
| --- | --- | --- |
| **Low certainty (low to very low certainty evidence)** | | |
| Category 0: might be not convincingly different than acupuncture | Amitriptyline | Very low |
|  | Amitriptylinoxide | Very low |
|  | Clomipramine | Very low |
|  | Doxepin | Very low |
|  | Imipramine | Very low |

**Footnote:** TCAs, tricyclic antidepressants; NMA, network meta-analysis. *Categories do not inform value judgements about the importance of the effects; **Certainty of evidence for each intervention when compared with acupuncture.

**eTable 8.** Final classification of TCAs and acupuncture, based on NMA of intervention for responder rate

| **Certainty of the evidence, and classification* of intervention** | **Intervention** | **Certainty of the evidence**** | |
| --- | --- | --- | --- |
| **Low certainty (low to very low certainty evidence)** | | |  |
| Category 0: might be not convincingly different than acupuncture | Amitriptyline | Very low | |
|  | Amitriptylinoxide | Very low | |

**Footnote:** TCAs, tricyclic antidepressants; NMA, network meta-analysis. *Categories do not inform value judgements about the importance of the effects. **Certainty of evidence for each intervention when compared with acupuncture.

**eTable 9.** Final classification of TCAs and acupuncture, based on NMA of intervention for AE rate

| **Certainty of the evidence, and classification* of intervention** | **Intervention** | **Certainty of the evidence**** |
| --- | --- | --- |
| **Low certainty (low to very low certainty evidence)** | | |
| Category 1: might be more harmful than acupuncture | Amitriptyline | Very low |
| Category 0: not convincingly different than acupuncture | Amitriptylinoxide | Very low |

**Footnote:** TCAs, tricyclic antidepressants; NMA, network meta-analysis; AE, adverse event. *Categories do not inform value judgements about the importance of the effects. **Certainty of evidence for each intervention when compared with acupuncture.

**eFigure 1.** Risk of bias of eligible RCTs


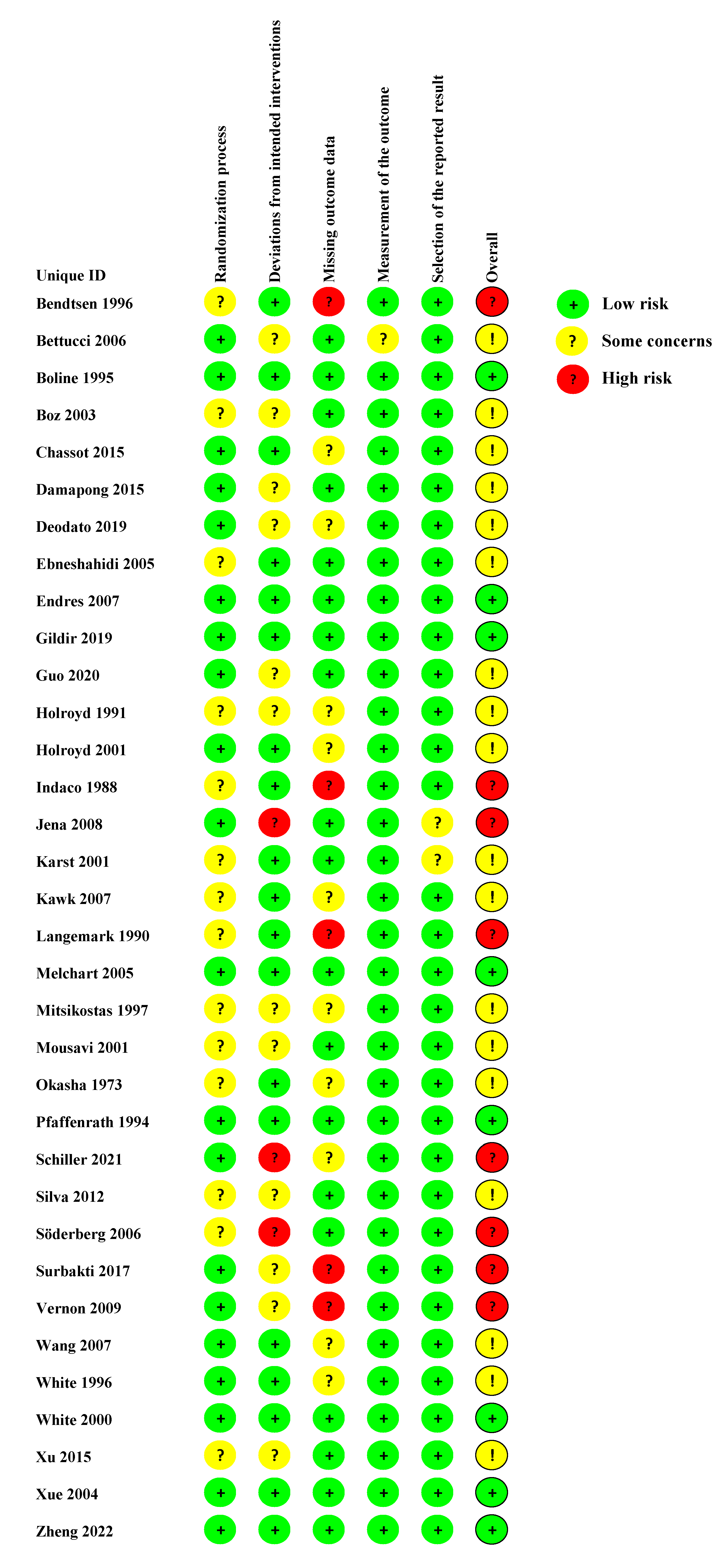


**eFigure 2.** The “dev-dev” plots of random-effects consistency and inconsistency models for NMA by outcomes


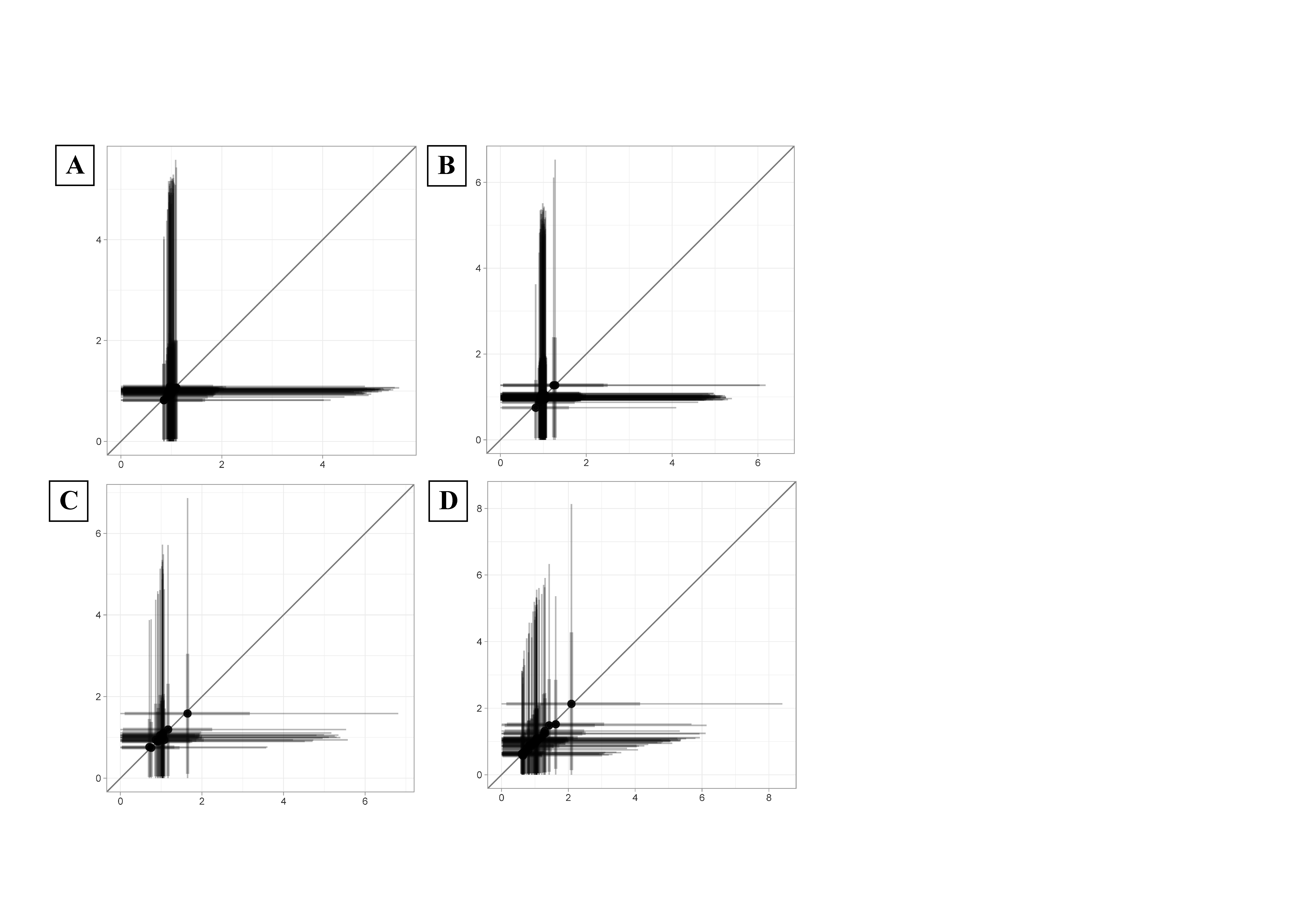


**Footnote:** NMA, network meta-analysis. A: headache frequency; B: headache intensity; C: responder rate; D: adverse event. The horizontal coordinate represents residual deviance of consistency model and the vertical coordinate represents residual deviance of inconsistency model. All points lie roughly on the line of equality, indicating that there is no evidence for inconsistency.

**eFigure 3.** Network diagram of comparison of headache frequency at a category-level


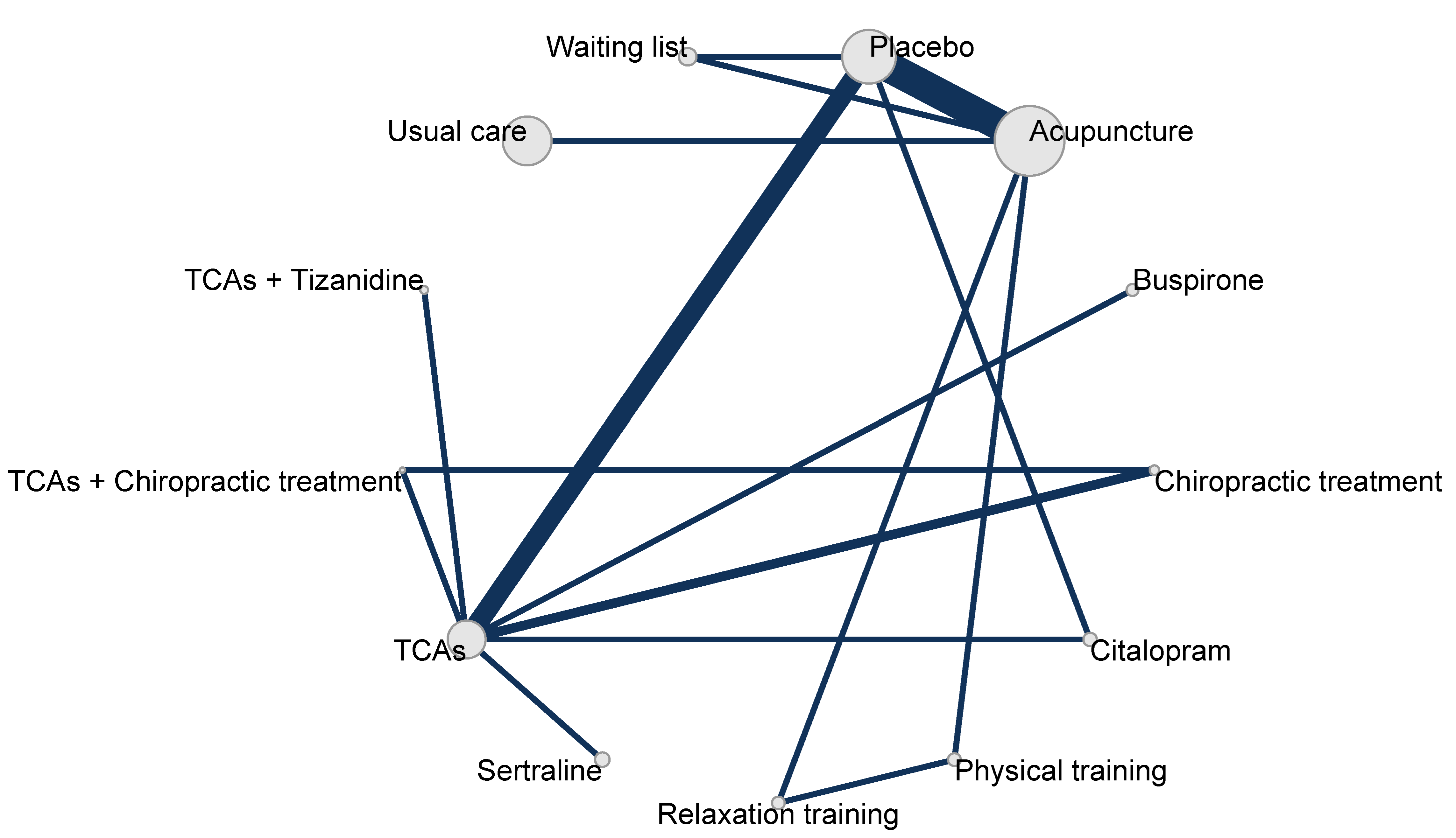


**Footnote:** TCAs, tricyclic antidepressants. The size of the grey nodes represents the number of included participants, and the thickness of the line represents the number of included studies.

**eFigure 4.** Network diagram of comparison of headache frequency at an individual-level


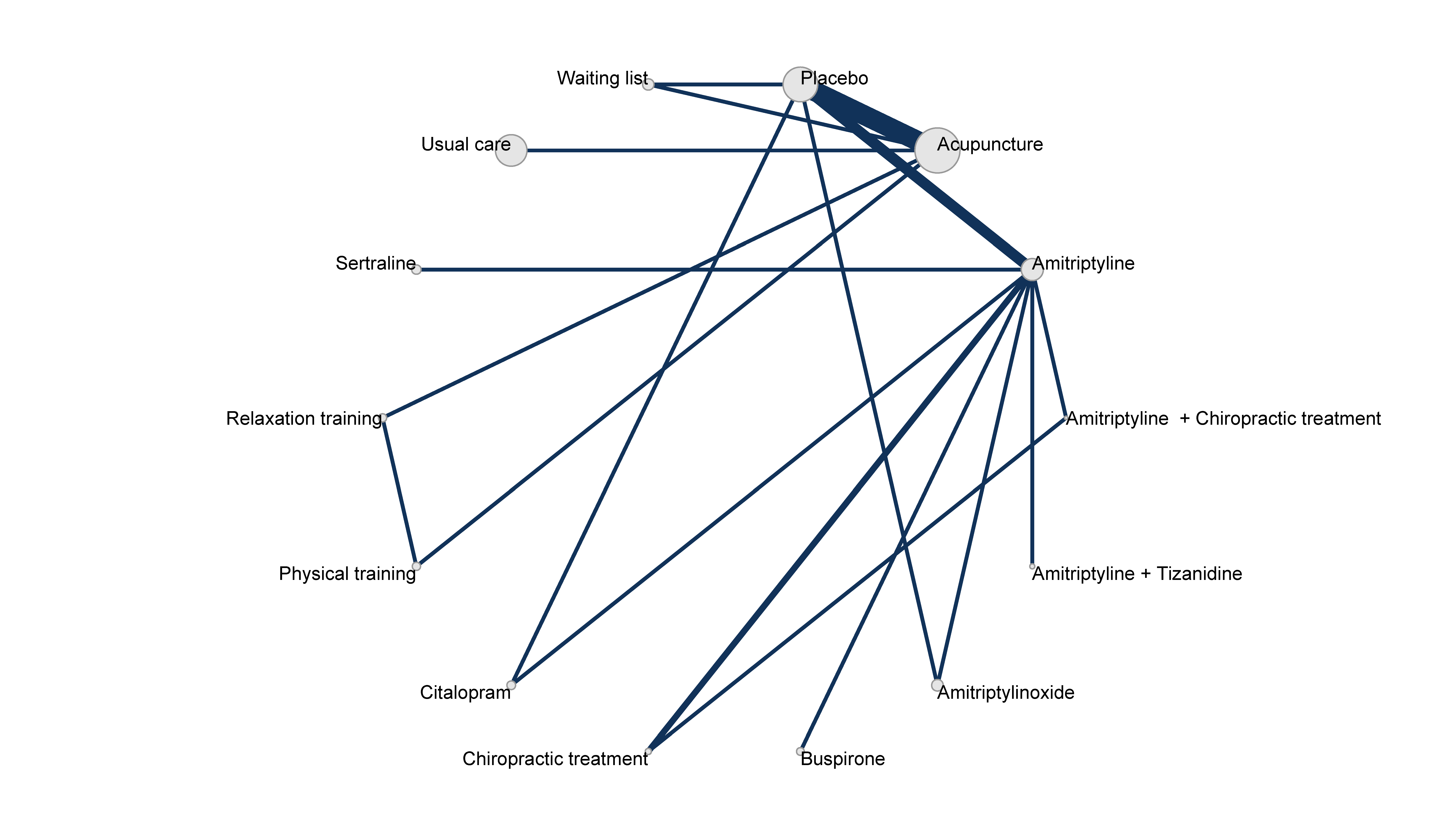


**Footnote:** The size of the grey nodes represents the number of included participants, and the thickness of the line represents the number of included studies.

**eFigure 5.** Network diagram of comparison of headache intensity at a category-level


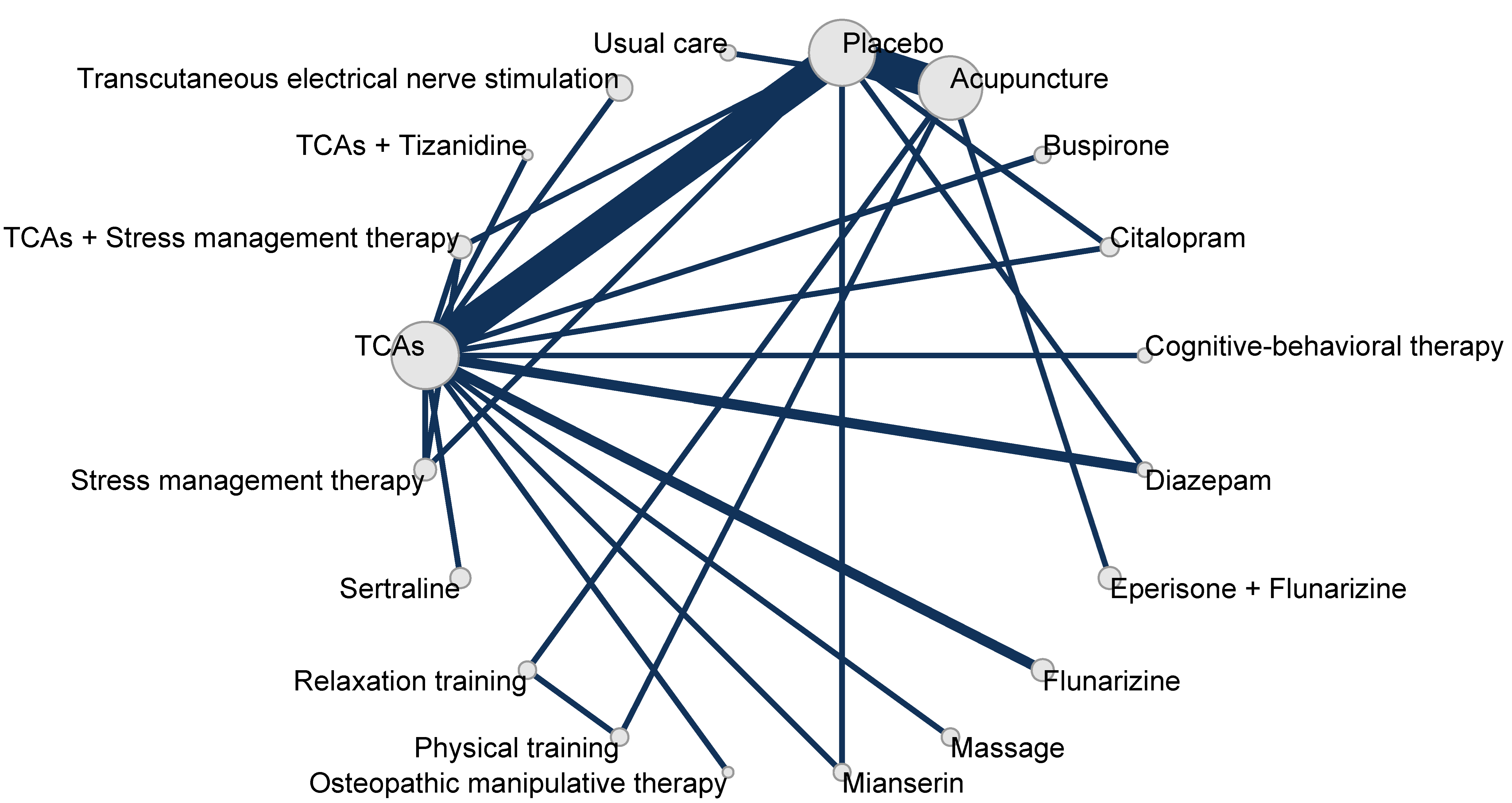


**Footnote:** TCAs, tricyclic antidepressants. The size of the grey nodes represents the number of included participants, and the thickness of the line represents the number of included studies.

**eFigure 6.** Network diagram of comparison of headache intensity at an individual-level


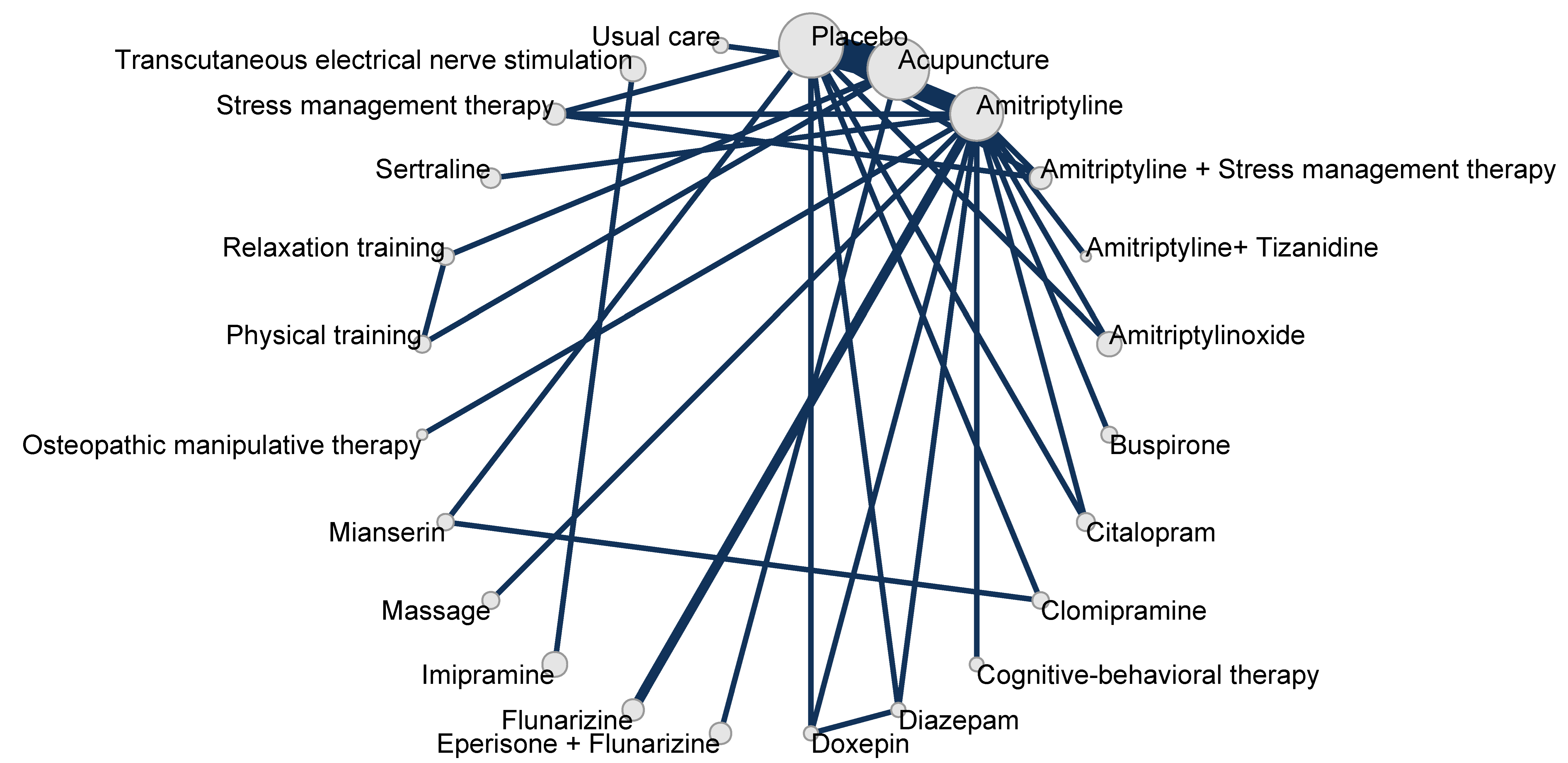


**Footnote:** The size of the grey nodes represents the number of included participants, and the thickness of the line represents the number of included studies.

**eFigure 7.** Estimate of comparison between acupuncture and TCAs of responder rate


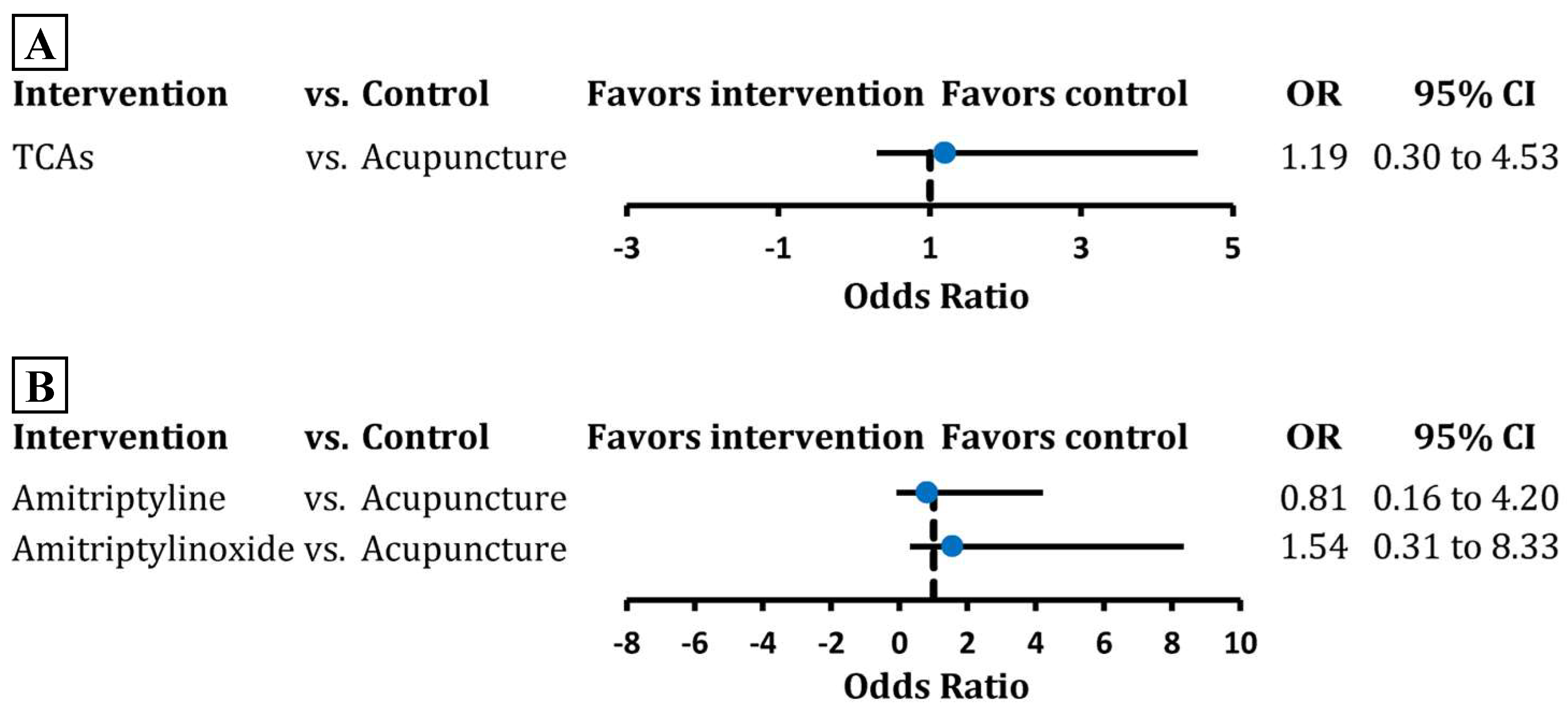


**Footnote:** TCAs, tricyclic antidepressants; OR, odds ratio; CI, confidence interval. The black vertical line corresponds to 1.

**eFigure 8.** Network diagram of comparison of responder rate at a category-level


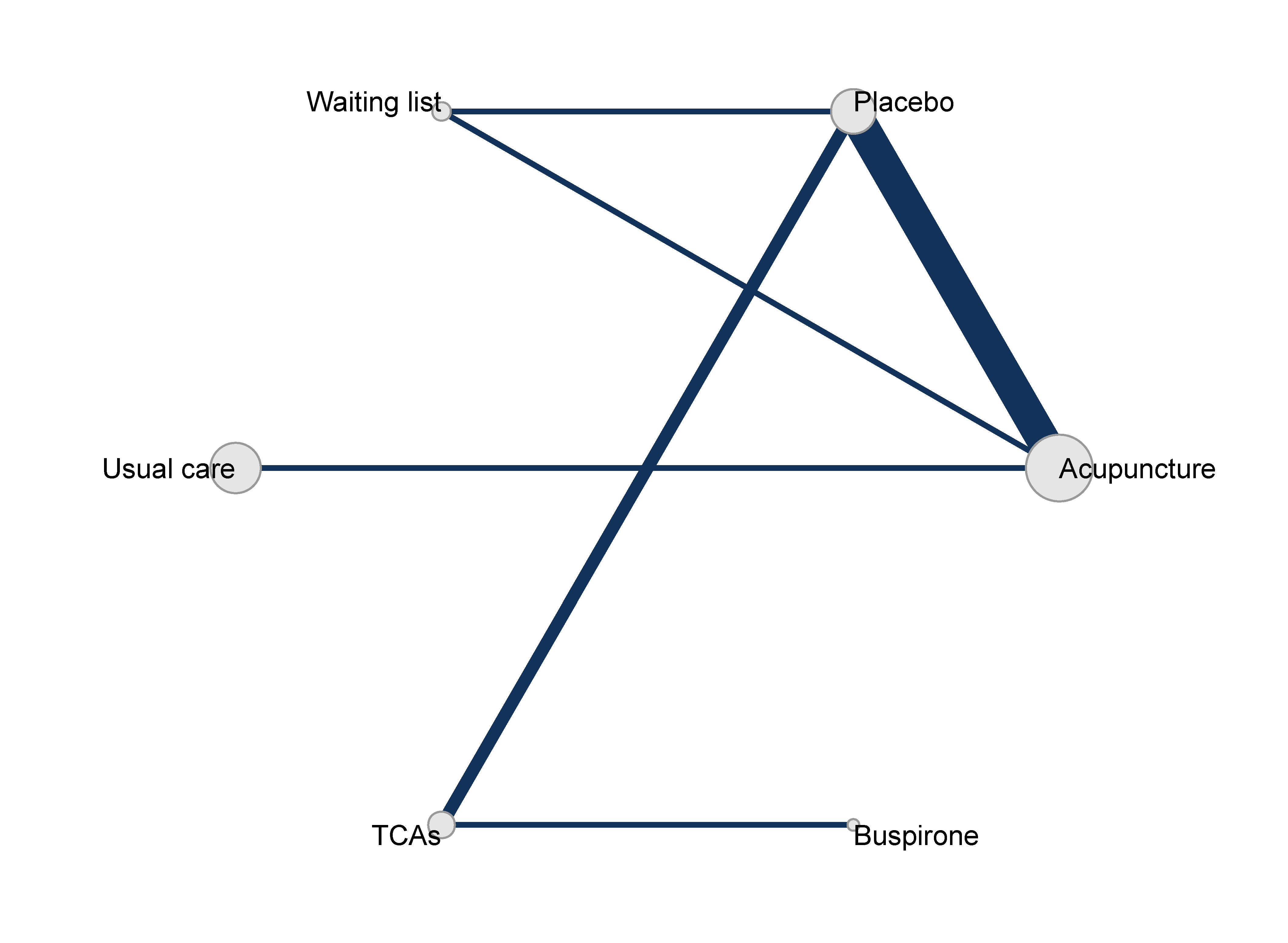


**Footnote:** TCAs, tricyclic antidepressants. The size of the grey nodes represents the number of included participants, and the thickness of the line represents the number of included studies.

**eFigure 9.** Network diagram of comparison of responder rate at an individual-level


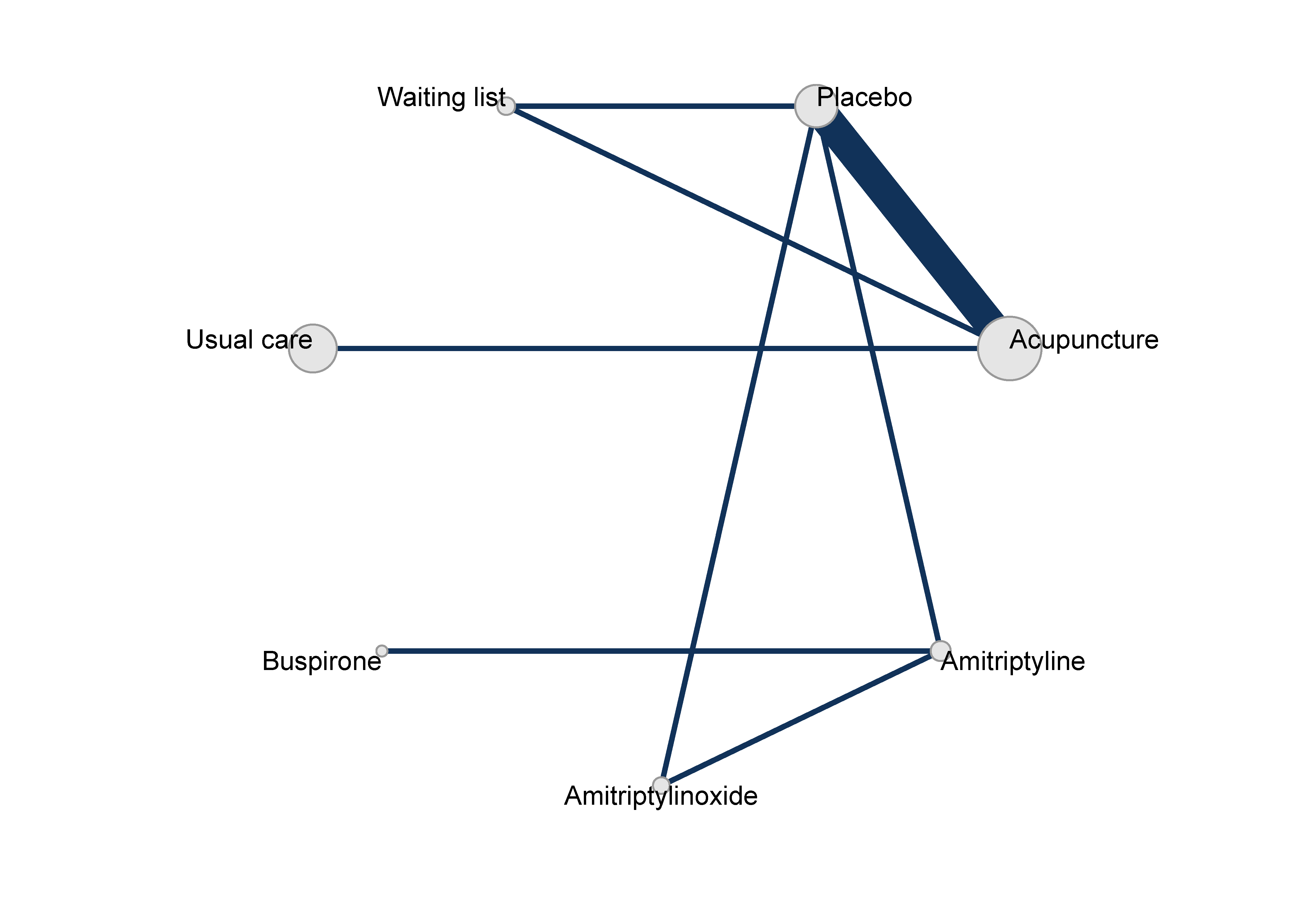


**Footnote:** The size of the grey nodes represents the number of included participants, and the thickness of the line represents the number of included studies.

**eFigure 10.** Estimate of comparison between acupuncture and TCAs of adverse event rate


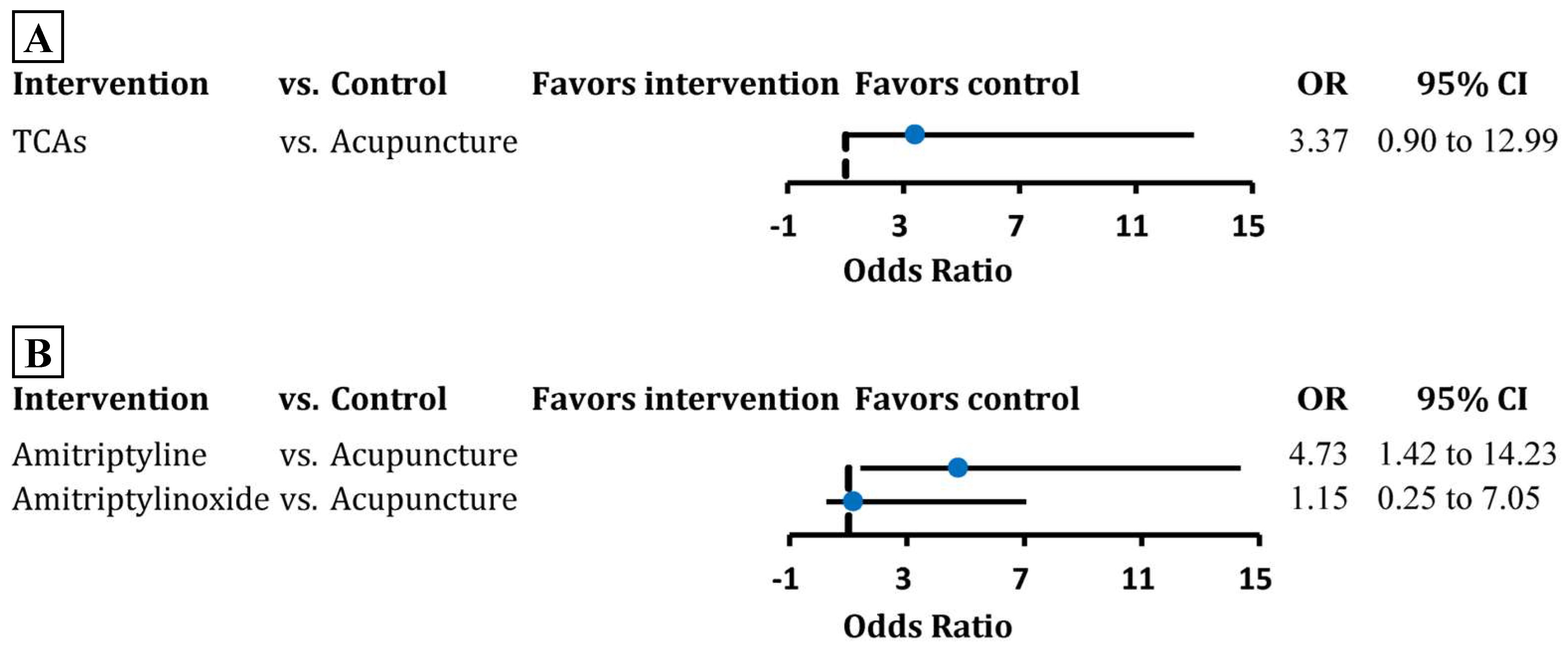


**Footnote:** TCAs, tricyclic antidepressants; OR, odds ratio; CI, confidence interval. The black vertical line corresponds to 1.

**eFigure 11.** Network diagram of comparison of adverse event rate at a category-level


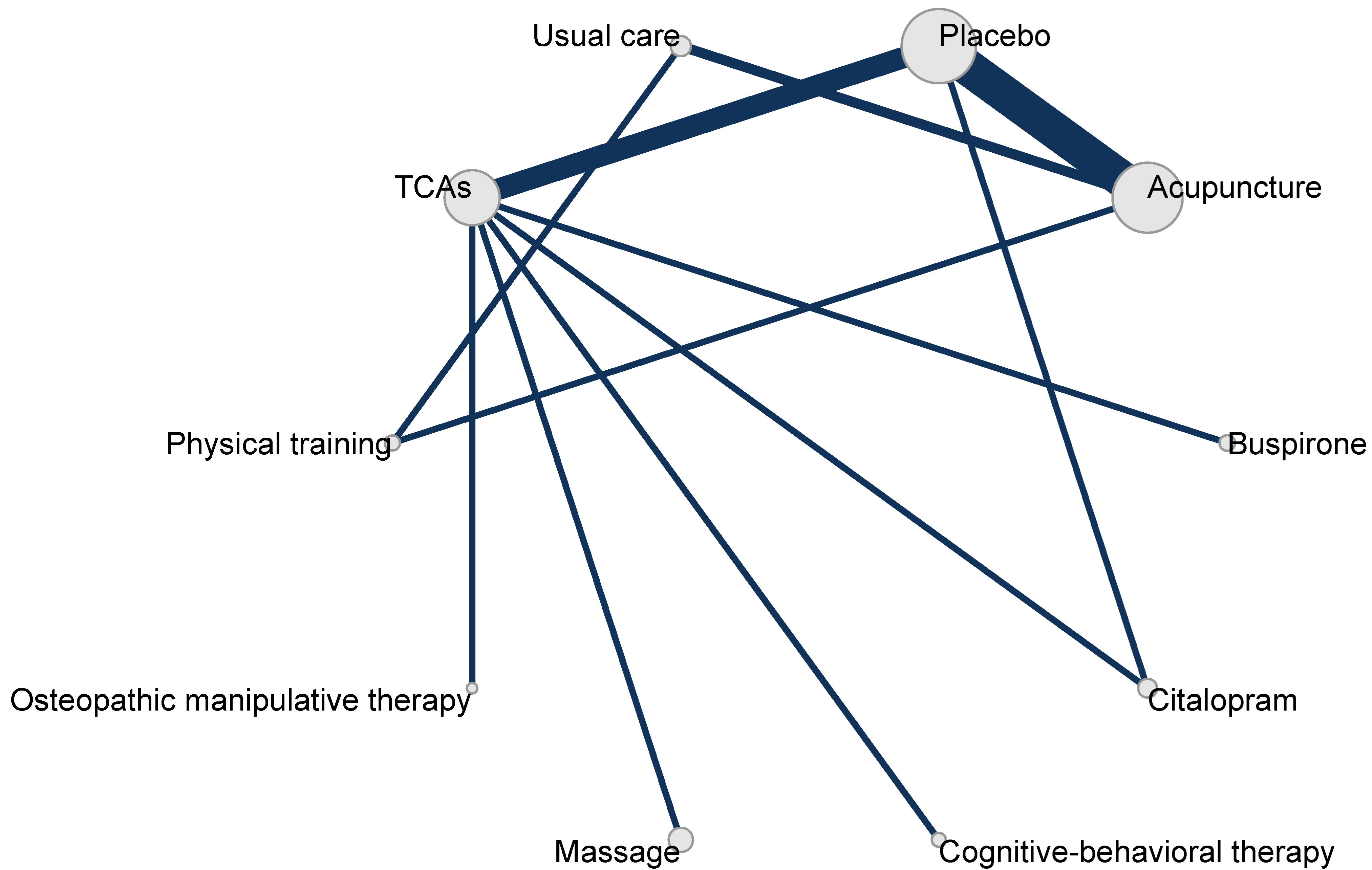


**Footnote:** TCAs, tricyclic antidepressants. The size of the grey nodes represents the number of included participants, and the thickness of the line represents the number of included studies.

**eFigure 12.** Network diagram of comparison of adverse event rate at an individual-level


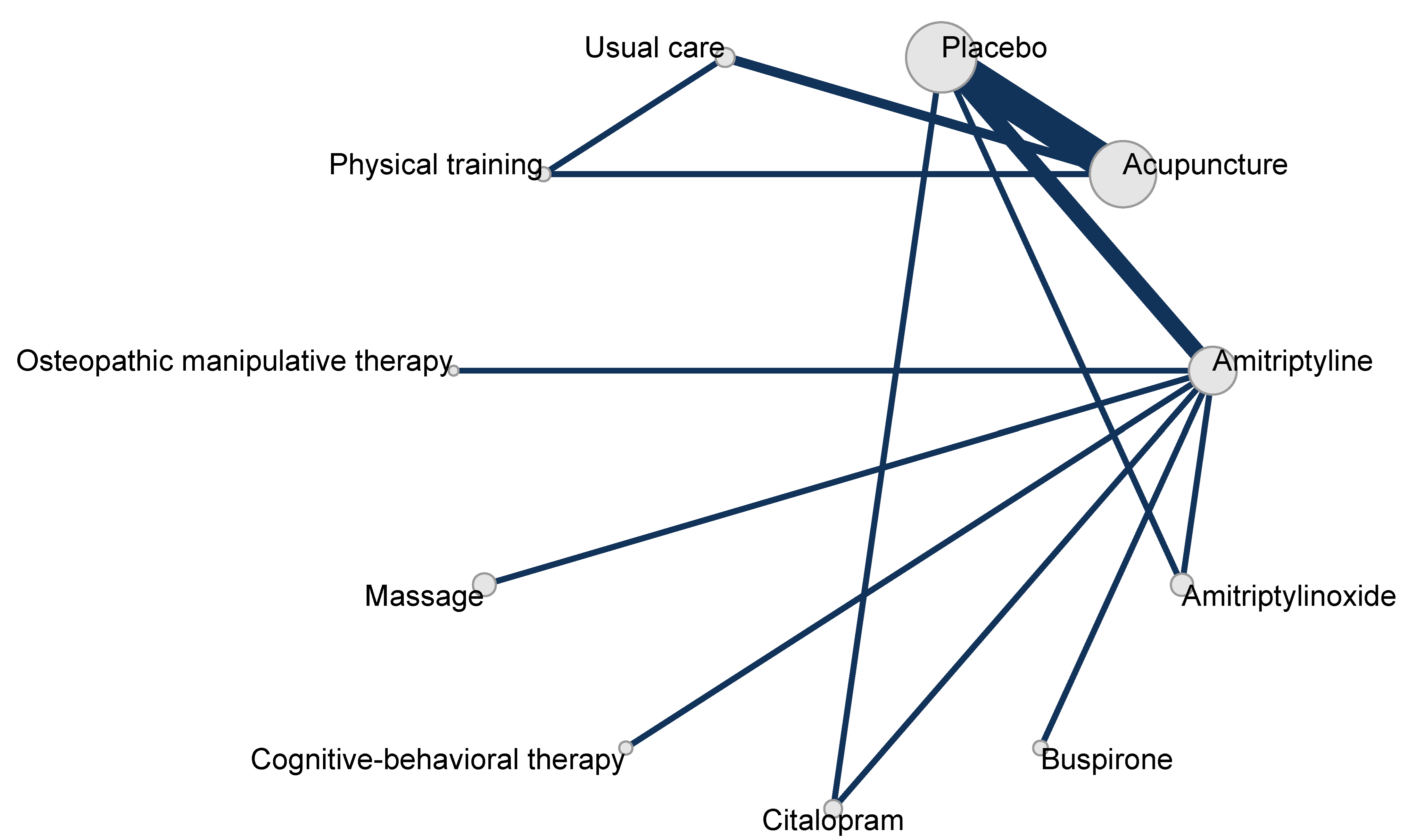


**Footnote:** The size of the grey nodes represents the number of included participants, and the thickness of the line represents the number of included studies.

**eFigure 13.** Sensitivity analysis of headache frequency after excluding RCTs at high risk of bias

**
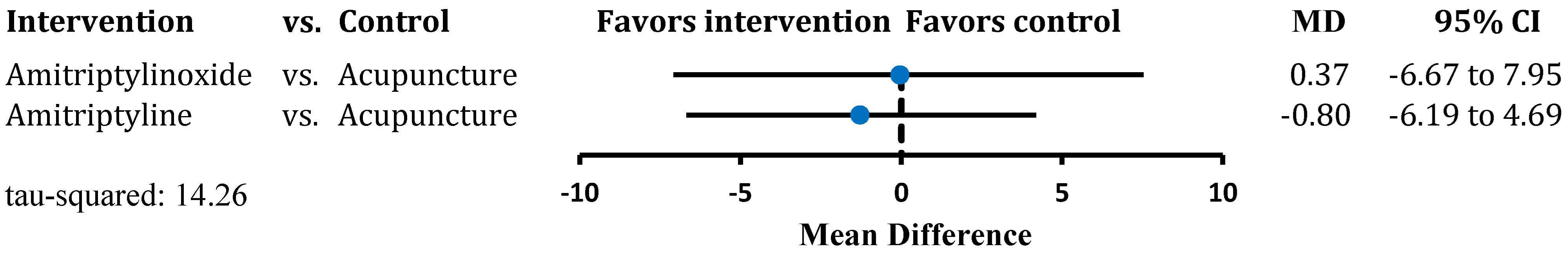
**

**Footnote:** RCT, randomized controlled trial; MD, mean difference; CI, confidence interval. The black vertical line corresponds to 0.

**eFigure 14.** Sensitivity analysis of headache frequency after excluding RCTs less than 50 participants

**
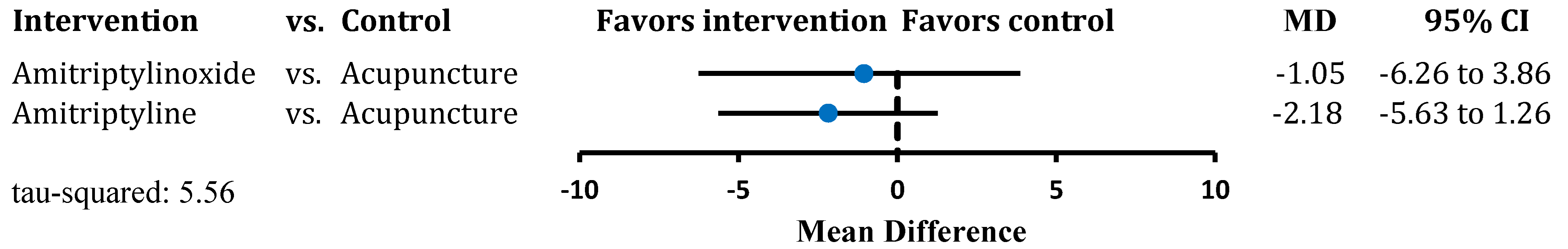
**

**Footnote:** RCT, randomized controlled trial; MD, mean difference; CI, confidence interval. The black vertical line corresponds to 0.

**eFigure 15.** Subgroup analysis of headache frequency on chronic tension-type headache


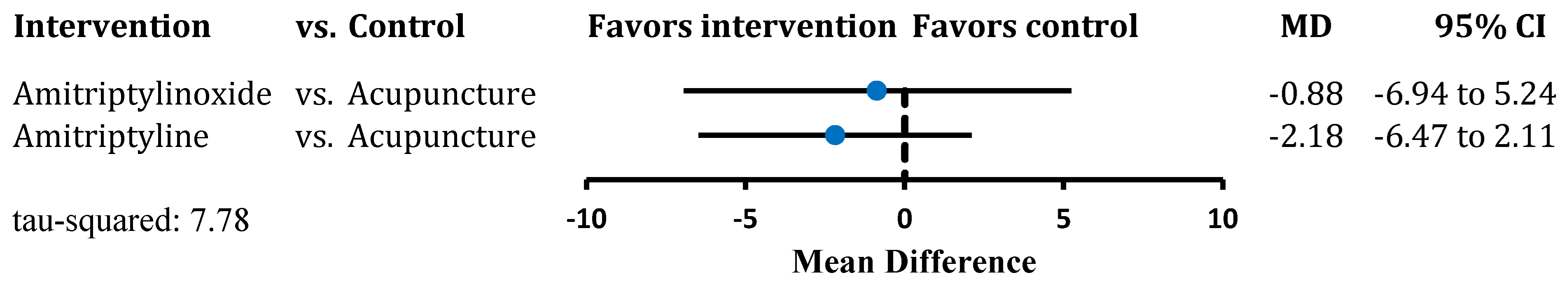


**Footnote:** MD, mean difference; CI, confidence interval. The black vertical line corresponds to 0.

**eFigure 16.** Subgroup analysis of headache frequency on different classification of acupuncture

**
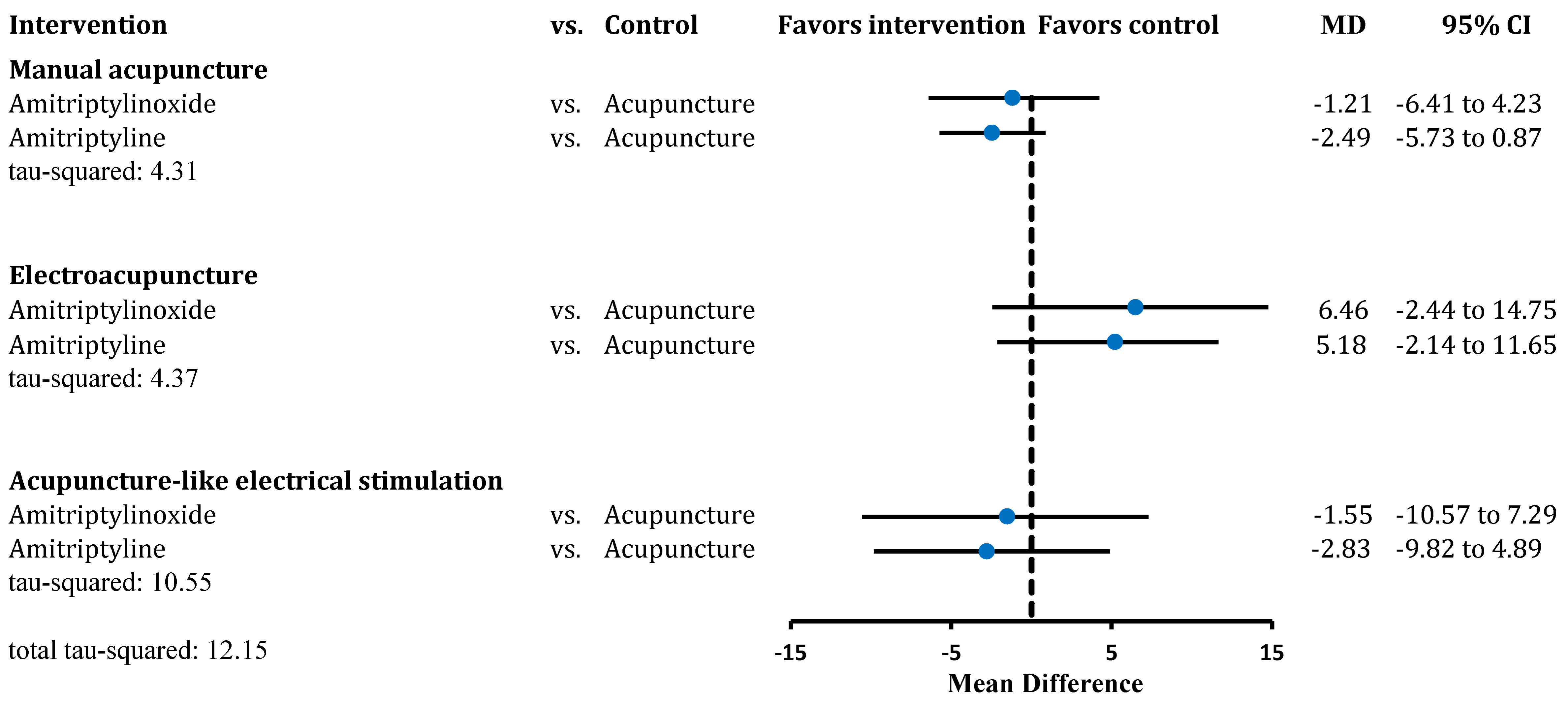
**

**Footnote:** MD, mean difference; CI, confidence interval. The black vertical line corresponds to 0.

**eFigure 17.** Subgroup analysis of headache frequency on different endpoint of treatment


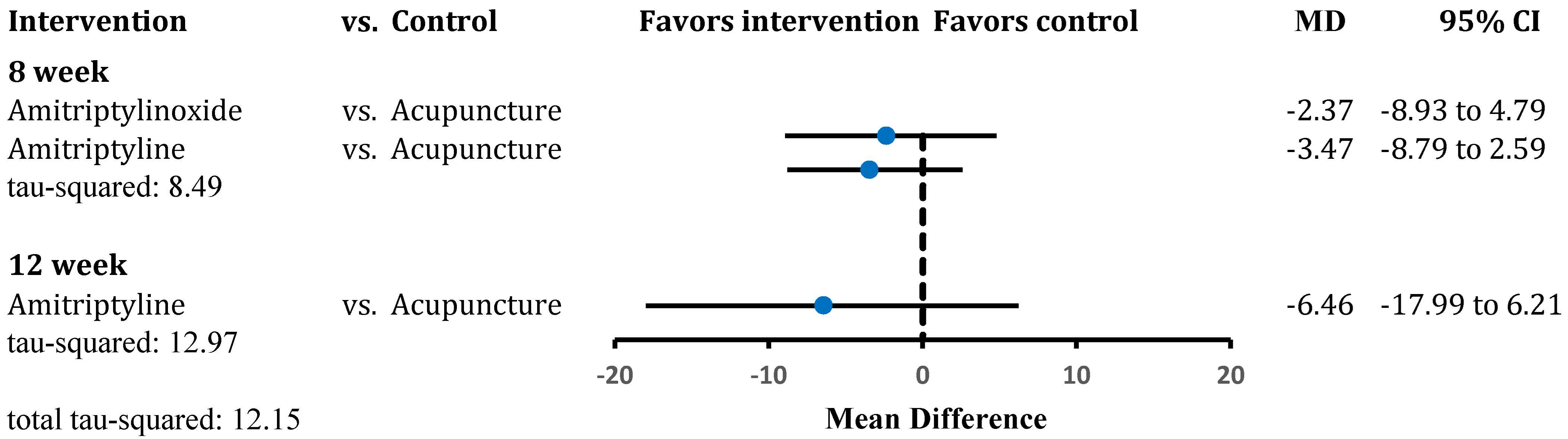


**Footnote:** MD, mean difference; CI, confidence interval. The black vertical line corresponds to 0.
